# Toward Advanced Quantitative Prediction of Missense Variant Effects

**DOI:** 10.3390/ijms27198777

**Published:** 2026-09-30

**Authors:** Shaopei Ye, Selen Özkan, Aitana Díaz-Vásquez, Natàlia Padilla, Xavier de la Cruz

**Affiliations:** 1 Research Unit in Clinical and Translational Bioinformatics, Vall d’Hebron Institute of Research (VHIR), Universitat Autònoma de Barcelona, Bellaterra, 08193 Barcelona, Spain; shaopeiye55@gmail.com (S.Y.); selenozkan23@gmail.com (S.Ö.); aitana.diaz@vhir.org (A.D.-V.); padilla.sirera@gmail.com (N.P.); 2 Institució Catalana de Recerca i Estudis Avançats (ICREA), 08010 Barcelona, Spain

**Keywords:** missense variants, variant effect prediction, mutation tolerance, molecular endophenotype, clinical variant interpretation, protein-specific predictors, two-step position-informed regression, ensemble learning

## Abstract

Predicting how missense variants alter protein function is central to resolving the genotype-to-phenotype equation, a task now addressed by numerous computational predictors. The most accurate are increasingly opaque, however, limiting both their mechanistic value and their adoption in the clinic. Here we present QAFIsplit, a redesign of the Quantitative Assessment of Functional Impact framework that models variant effect in two interpretable stages: the mutation tolerance of a position, and the deviation introduced by a specific substitution. Framed as the prediction of a molecular endophenotype, QAFIsplit reaches state-of-the-art quantitative accuracy on independent deep mutational scanning benchmarks, surpassing black-box predictors such as AlphaMissense in most protein domains. Carried into clinical variant classification, it performs on par with established clinical tools without ever being trained on clinical labels, and complements meta-predictors without penalty. In the CAGI 7 community experiment, QAFI-based strategies were submitted blindly to four independent disease-gene challenges, backed by strong pre-submission clinical validation (AUC up to 0.99).

## 1. Introduction

In their influential review, Shendure et al. [1] proposed that advancing genomic medicine requires “pivoting back to basics”—focusing on solving the genotype-to-phenotype equation. Central to their proposed roadmap (their Figure 4) is the development of algorithms to predict the functional impact of genetic variants, a prerequisite for both genetic diagnosis and pharmacogenomics. Because disentangling the full genotype-to-phenotype map—from DNA to disease—remains out of reach, a productive intermediate step is to target the molecular endophenotype: a quantitative, biochemical description of how sequence changes alter protein function [2]. This approach assumes that accurately mapping this molecular layer is a prerequisite for ultimately resolving clinical phenotypes.

The field has increasingly pursued this through data-driven machine learning, a trajectory fueled by the growing availability of large-scale functional data such as deep mutational scanning (DMS) assays [3,4,5,6], now systematically collected in dedicated repositories. This methodological progression has moved from early regression models [7,8] and generative evolutionary models like EVE [9], to protein language models [10,11] and structure-informed predictors like AlphaMissense [12], with the most recent efforts extending to genome-wide language models [13]. While this has expanded predictive scale, it has come at a cost. As models have grown more powerful, they have become opaque black boxes, resisting the mechanistic inspection required for clinical adoption [14].

Furthermore, translating these continuous molecular endophenotypes into clinical utility faces a distinct challenge. Clinical genetics still relies heavily on binary pathogenic/benign classifications, and the binary classifiers used today (e.g., REVEL [15], BayesDel [16]) have become established enough to serve as evidence under ACMG/AMP guidelines [17,18,19]. Yet their true predictive capacity is hard to establish: because these tools are widely used to generate the very clinical labels against which they are later benchmarked, a degree of circularity inflates and obscures their apparent performance [20]. Continuous, mechanistically grounded models offer an alternative that is biologically more realistic and conceptually easier to generalize to other biological layers. Their utility for clinical genetics, however, hinges on a prerequisite: demonstrating that these molecular scores can bridge the gap to clinical reality.

Here, we address this by proposing a framework to model the molecular endophenotype through a fundamental redesign of the Quantitative Assessment of Functional Impact (QAFI) architecture. Building on QAFI 1.0 [21], we reformulate variant effect prediction as a two-stage problem (Figure 1). First, we estimate the mutation tolerance of a position—defined as the median functional effect across all possible substitutions—reflecting whether the local structural environment is inherently fragile or robust. Second, we model the deviation of individual variants from that positional baseline. This decomposition preserves interpretability at the biological level, even when utilizing non-linear learning via XGBoost.

This work is structured around two goals. First, we establish QAFIsplit as a competitive, mechanistically grounded predictor of the molecular endophenotype. Second, we ask how far our endophenotype predictions carry into clinical classification—a test of the genotype-to-phenotype conjecture at the accuracy we currently reach.

In this work, we demonstrate that:

(i) The two-stage QAFIsplit reformulation provides a biophysically grounded architecture that outperforms general-purpose predictors on independent functional benchmarks—surpassing AlphaMissense in 86% of tested protein domains.
(ii) Carried into clinical variant classification, this molecular-level performance yields an AUC of 0.90 (on par with AlphaMissense), nearly reaching the limit set by its feature representation despite never being trained on clinical data, and it integrates into high-performing meta-predictors without loss of performance, enabling hybrid strategies with a stronger biophysical grounding.
(iii) Under fully blind conditions, both the stand-alone predictor and the meta-predictor strategies showed strong performance across four independent CAGI 7 challenges (ATP7B, LPL, ARSA, and TSC2)—supporting their generalization to entirely unseen proteins.

## 2. Results

In the following sections, we first describe in more detail our new prediction tool, and then we go through the different training and validation steps followed to establish its validity.

Tables S1 and S2 provide a concise overview of all the models described and the relationships among them.

### 2.1. A Two-Stage Framework for Quantitative Variant Effect Prediction

In our new model of the impact of missense variants on protein function, we have given a central role to mutation tolerance. Operationally, this property is defined as the median functional effect across all 19 possible amino acid substitutions at a specific position in the protein sequence. It reflects the inherent sensitivity of a position to mutation, and its predicted value may be a relevant component for estimating the impact of specific mutations.

At the technical level, this view translates into a reformulation of the prediction problem into two distinct steps (Figure 1): first, estimating the mutation tolerance of a protein position, and second, using this information, combined with other features, to predict the impact of individual variants at that position.

Both steps employ the same ensemble learning strategy, where predictions from multiple protein-specific models (PSPs) are aggregated to produce the final output. The nature of these PSPs, trained using protein-specific DMS data from the literature, varies depending on the predictive target (mutation tolerance or variant effect). The resulting methodology keeps the name of our original predictor (QAFI, here referred to as QAFI 1.0) [21], although a “split” suffix has been added to the name, QAFIsplit, to indicate the two main predictive steps.

The technical implementation of this design has yielded three versions of QAFIsplit—QAFIsplit 1.0, QAFIsplit 2.0, and QAFIsplit 3.0—which differ in how we solved the variant effect step. A graphical overview of the development process is shown in Figure S1.

In the following sections, we first describe the cross-validated performance of the protein-specific models (PSPs) for each of the two predictive tasks separately—mutation tolerance (Section 2.2.1) and variant effect (Section 2.2.2). We then describe the ensemble strategy and its performance as a unified framework (Section 2.3), followed by an extensive external validation and benchmarking of the resulting models (Section 2.4).

### 2.2. The Building Blocks of Our Methodology: PSPs

In the following subsections, we describe how the PSPs for mutation tolerance (mtPSPs) and variant effect (vePSPs) prediction performed. PSPs form the foundation of the QAFI framework: their within-protein performance defines the upper bound for the ensemble models described in subsequent sections.

#### 2.2.1. Predicting Mutation Tolerance (mtPSPs)

mtPSPs are protein-specific models that estimate positional mutation tolerance and are trained on median effects derived from deep mutational scanning (DMS) data (see Materials and Methods). Each mtPSP is a multiple linear regression on ten position-level descriptors: three derived from the multiple sequence alignment and seven computed from the protein’s AlphaFold model. Once fitted, it returns a tolerance value for any position of the protein, including positions with no experimental measurement. Full details are given in Section 4.11 and Table S6; Table S1 summarizes the key developmental decisions.

Across the 60 proteins, predicted mutation tolerances showed good agreement with experimental values (Figure 2 and Figure S2). Visual inspection of observed-versus-predicted plots (Figure 2A) shows that the data tend to align with the diagonal, although with protein-dependent patterns. For example, some mtPSPs tended to overestimate tolerance near the lower end of the observed range (Figure 2A).

The observed–predicted Pearson (Figure 2B) and Spearman (Figure S3) correlations confirm the visual analysis, providing a quantitative assessment of performance variability across proteins. A clear trend emerges: this variability is lower-bounded, as for more than two-thirds of the proteins both correlation measures exceed 0.4, with several reaching above 0.8. Together, these results support the predictive value of the selected features and the robustness of the mutation-tolerance models.

To illustrate performance beyond summary metrics, we examine how it reproduces observed position-specific patterns along the protein sequence, using PRKN as a high-performing example (Figure S4; r = 0.85). Predicted tolerances closely track the observed variations across the sequence, largely independent of annotated domain boundaries. Notably, our method accurately captures the plateau of high experimental mutation tolerance corresponding to a known disordered region in PRKN [22]. This behavior is most likely due to the inclusion, among the model’s predictive features, of AlphaFold pLDDT [23], whose low values are associated with protein disorder [24].

A comparable correspondence between predicted and experimental mutation tolerance profiles is observed across additional proteins in our dataset, with its extent depending on the correlation level (Figure S5). Overall, the model captures global trends and, in higher-correlation cases, also local variations in experimental positional medians along the protein sequence. Mutation tolerance peaks are usually well located, although their predicted size tends to be smaller than their actual size. Together, these results indicate that our predictions preserve meaningful position-specific signals across a broad range of proteins, extending beyond individual high-performing examples.

#### 2.2.2. Predicting the Variant Effect (vePSPs)

The vePSP models estimate variant functional impact using two PSP model versions, PSPsplit 1.0 and PSPsplit 2.0, which share common input data and feature sets but differ in their training targets. As a reference, we use two baseline models, PSP 1.0 and PSP 2.0. The former corresponds to our original QAFI model, and the second is an upgrade using the full set of predictive features presented here. A summary of the training details and model specifications for all these tools is provided in Table S1.

Model performance was evaluated by comparing observed and predicted variant-level functional scores, illustrated first using VKORC1 as an example (Figure 3A). In this case, we see how the split-based models shift the main cluster of variants closer to the diagonal relative to the baseline approaches, resulting in higher correlation coefficients. For the set of 60 proteins, these trends were summarized using Pearson and Spearman correlations together with prediction errors (MAE and RMSE) (Figure 3B,C and Figure S6).

Examination of these results reveals several clear trends. First, among the baseline models, PSP 2.0 consistently outperformed PSP 1.0, demonstrating the added value of the expanded feature set and the non-linear regression model (Figure 3B). Turning to the split-based approaches, PSPsplit 1.0 achieved improved performance over PSP 2.0 for most proteins, confirming that incorporating mutation-tolerance estimates can enhance variant-level prediction. By contrast, PSPsplit 2.0 did not show a systematic advantage: for most proteins, both PSP 2.0 and PSPsplit 1.0 performed better, indicating that the PSPsplit 2.0 strategy did not confer consistent improvement.

All models reduced prediction errors relative to PSP 1.0, with PSP 2.0, PSPsplit 1.0, and PSPsplit 2.0 showing comparable absolute error levels, as further confirmed by MAE and RMSE analyses across all 60 proteins (Figure S6). Together, these results indicate that the main performance gain derives from incorporating mutation tolerance as an intermediate feature.

### 2.3. Proteome-Wide Quantitative Variant Effect Prediction: The QAFI Ensemble Framework

It is a priori unclear whether the observed PSP-level gains translate to the ensemble level: PSPs may improve by capturing protein-specific effects that are not transferable across proteins, resulting in performance loss upon aggregation. In this section, we examine whether and how these gains map to QAFI ensemble performance.

#### 2.3.1. Defining the QAFI Ensemble Framework

The framework rests on two choices: how many protein-specific predictors (PSPs) the ensemble contains (its size, N) and which proteins they correspond to. Both are determined jointly by a nested leave-one-protein-out procedure that selects, for a given N, the PSPs generalizing most consistently across proteins (Figure S7A; full procedure in Methods).

We estimated performance by cross-validation, holding out each protein in turn and predicting its variants from an ensemble built only from the others—so that every protein is scored as if it were new. Across the 60 proteins, we tested five ensemble sizes (*N* = 10, 15, 30, 45, and 60) and selected the one performing best in most proteins, *N* = 30 (Figure S7B,C); the corresponding proteins are listed in Table S2.

#### 2.3.2. Cross-Validated Performance of the QAFI Ensemble Framework

The ensemble strategy described in Section 2.3.1 was applied separately to the different PSP families (see Table S3 for the mapping between QAFI models and PSPs), resulting in three main QAFI models: **QAFI 2.0** (based on PSP 2.0), **QAFIsplit 1.0** (based on PSPsplit 1.0), and **QAFIsplit 2.0** (based on PSPsplit 2.0). In addition, we introduced a fourth model, **QAFIsplit 3.0**, derived from PSPsplit 2.0 but using a correlation-weighted fusion scheme (Figure S1B) instead of the median for aggregation. For each PSP–QAFI pair (e.g., PSPsplit 1.0 vs. QAFIsplit 1.0), we compared model performance separately for the 60 proteins in our dataset, to assess the performance deviation introduced by moving from within-protein to cross-protein prediction and to estimate the generalization capacity of the ensemble framework.

In all cases, data points clustered close to, but predominantly above, the diagonal (Figure 4A and Figure S8), indicating that ensemble models largely preserve the performance trends of their underlying PSPs, although a generalization gap is observed. SNCA (α-synuclein) emerged as a consistent outlier showing a marked PSP–QAFI discrepancy across models, consistent with its intrinsically disordered nature [25], which contrasts with the predominantly globular character of the proteins whose PSPs define the ensemble.

When compared with the original QAFI 1.0, all new ensemble models showed an overall performance gain (Figure 4B and Figure S9), demonstrating that the improvements observed at the PSP level translated effectively to the ensemble setting. Differences among QAFIsplit models and QAFI 2.0 were generally minor in absolute terms; their consistency is examined on independent data in Section 2.4.1.

### 2.4. External Validation and Benchmarking of QAFI Models

The cross-validated results presented in the previous section provide an internal assessment of model performance. Here we go further, evaluating the QAFI framework on entirely independent data not involved in any stage of model development. This external validation comprises three complementary analyses: a large-scale benchmark across 402 human protein domains, an evaluation on clinically annotated variants, and performance assessment in four CAGI 7 challenges (TSC2, ATP7B, ARSA, and LPL). Each analysis also serves as an opportunity to benchmark QAFI against state-of-the-art predictors in the field.

#### 2.4.1. Large-Scale Validation Across 402 Human Protein Domains

The large-scale site-saturation mutagenesis dataset recently published by Beltrán et al. [26] provides experimental measurements across an independent collection of human protein domains. The size and technical homogeneity of this dataset, together with the diversity of protein domains covered, make it an important validation resource.

After removing any potential overlap with the development set, the benchmark comprised 492,096 missense substitutions across 402 protein domains. Using this benchmark to compare QAFI family members, we observe that QAFIsplit 3.0 outperforms the remaining members of the QAFI family in the majority of domains, ranking above QAFI 1.0 (87%, *p* = 1.1 × 10^−52^), QAFI 2.0 (71%, *p* = 8.1 × 10^−18^), QAFIsplit 1.0 (67%, *p* = 1.8 × 10^−11^), and QAFIsplit 2.0 (61%, *p* = 1.1 × 10^−5^) of cases (Figure S10). Model performance depends on the amount of experimental data available, but the relative ordering of the QAFI versions is preserved across domain length and number of measured variants (Figure S12).

We next benchmarked QAFIsplit 3.0 against a representative panel of external predictors (Figure 5). Overall, QAFIsplit 3.0 outperformed all general-purpose methods. It also performed at the level of the specialized versions of EVE and ESM1v, which were tailored to the characteristics of the domains in Beltrán et al.’s experiment: without any adaptation of its own, QAFIsplit 3.0 matched EVE_domain (49% of domains, *p* = 0.69) and remained close to ESM1v_domain (45%, *p* = 0.04). For thermoMPNN, given the substantial overlap between its training data and the Beltrán benchmark, the only dedicated stability predictor in the panel, we restricted the comparison to the homolog-free subset used by the original authors. In this setting, QAFIsplit 3.0 matched thermoMPNN’s performance (51% of domains).

Because Pearson correlation is insensitive to scale, we also compared the methods in terms of their deviation from the experimental values (Figure S14). On the raw outputs (Figure S14A), QAFIsplit 3.0 is the only method predicting within the range of the normalized functional assay, so its output can be used directly as a quantitative estimate of variant effect. After min–max-rescaling each method within each domain, QAFIsplit 3.0 still shows the lowest median deviation of all methods compared, with the exception of the domain-adapted version of ESM1v, which matches it (Figure S14B).

An interesting feature of QAFIsplit 3.0 predictions is that they tend to compensate for poor performance of other methods: there is a tail extending towards the right of the distribution, corresponding to proteins where the alternative method performed poorly while QAFIsplit 3.0 did not. This feature supports the idea that, alongside its potential as a stand-alone predictor, QAFIsplit 3.0 could also be used to complement existing in silico tools.

The variant-level success of QAFIsplit 3.0 is underpinned by the ensemble estimation of positional mutation tolerance (QAFImt). Figure S11 illustrates the performance of this first stage across the independent Beltrán et al. dataset. The radial plot (right panel) maps the per-protein Pearson correlations between predicted and observed positional medians for all protein domains, revealing a heterogeneous landscape. Less than 20% of domains exhibit poor agreement (*r* < 0.4), and more than 50% achieve good to excellent accuracy (*r* > 0.6). The representative sequence profiles (left panels) exemplify domains from this upper tier, demonstrating that when the baseline is well-estimated, QAFImt successfully captures the major trends and local variations in biophysical constraints. While the model struggles with certain protein contexts, this distribution confirms that the split architecture is nonetheless driven by a broadly reliable positional baseline for the majority of the proteome.

#### 2.4.2. Benchmarking QAFI on Clinically Annotated Variants

Here we ask how far our endophenotype models—our route to the genotype-to-phenotype problem—carry, at their present accuracy, into the clinic. As a test, we evaluated QAFIsplit 3.0 against a spectrum of established predictors on a common set of 12,787 clinically annotated variants (Figure 6).

To interpret QAFIsplit 3.0’s clinical coherence, we organized these methods into three reference groups. The first consists of established tools developed for clinical variant interpretation, many of which have been calibrated for use under ACMG/AMP guidelines (see Methods). This clinical ecosystem spans a remarkably broad performance range—from GERP++ (0.68), MPC (0.70) and PrimateAI (0.74) at the lower end, through PhyloP and FATHMM (0.77–0.78), SIFT (0.81), and CADD and PolyPhen (0.87), up to MutPred2 (0.93), REVEL and VEST4 (0.94), ClinPred (0.95) and BayesDel (0.96) at the top. Against this frame, QAFIsplit 3.0 reached an AUC of 0.90: without ever seeing a clinical label, it outperforms most of these tools and sits well above the median of the ecosystem.

The second reference indicates how much clinically relevant signal our feature representation can capture when optimized for classification. We estimate it with QAFIclas (0.92), an XGBoost classifier built on the same features as QAFIsplit 3.0 but trained directly on clinical labels. QAFIsplit 3.0 approaches this reference even though it never sees a clinical label.

The third group consists of advanced machine learning methods—such as CPT-1 (0.93), AlphaMissense (0.91) and EVE (0.89)—which, like QAFI, are not trained on clinical labels, though some have since been calibrated for clinical use [19]. QAFIsplit 3.0 falls within the range of this group, which delimits what can currently be achieved by predicting variant impact from molecular data alone.

Taken together, these comparisons place the endophenotype route within reach of the tools built directly on clinical data. Applicability was further supported by combining QAFIsplit 3.0 with AlphaMissense, popEVE, CPT-1, REVEL and BayesDel by median aggregation (QAFIMeta), which gave an AUC of 0.95 against 0.94 for the same combination without it: a continuous, biophysically grounded score integrates into established meta-predictors without penalty, forming the basis for the hybrid strategies we fielded in CAGI 7 (Section 2.4.3).

#### 2.4.3. External Blind Validation Through the CAGI 7 Community Experiment

To extend the independent validation of our methodology, we participated in the seventh edition of the Critical Assessment of Genome Interpretation (CAGI 7) community experiment (December 2025). CAGI provides an unbiased evaluation framework in which participants submit predictions without access to experimental outcomes, subsequently assessed by independent expert panels [27]. We focused on four protein-centric challenges involving missense variants measured through high-throughput functional or stability assays: ATP7B (Wilson disease, *n* = 15,795), LPL (familial lipoprotein lipase deficiency, *n* = 19,138), ARSA (metachromatic leukodystrophy, *n* = 8867), and TSC2 (tuberous sclerosis complex, *n* = 8891). Since our methodology is designed exclusively for missense variants, predictions were restricted to this subset in all four challenges. For all these challenges, we took advantage of CAGI guidelines that allow participants to submit up to six predictors, identifying one of them as their method of choice.

At the time of writing, the final challenge rankings have not been formally published; however, communications from the CAGI 7 organizers confirmed that our preferred models consistently ranked among the leading methods. Pending formal publication of these results, we describe here how we selected, among members of the QAFI family, our six candidates per challenge. The selection process was primarily evidence-based but with a qualitative integration step, as limited protein-specific data were available for fully automated ranking. It combined the performance of our models on the Beltrán et al. [26] dataset with an ROC analysis based on ClinVar-annotated variants for each challenge protein (Figure 7). The six models selected per challenge are shown in Figure 7, and a brief description of each is provided in Table S3.

Pre-submission evaluation of our models on ClinVar-annotated variants for the four challenge proteins confirmed strong discriminative performance across all challenges (Figure 7). AUC values were particularly high for LPL (0.978–0.998) and ATP7B (0.904–0.931), with solid performance also for ARSA (0.871–0.899) and TSC2 (0.868–0.922). These results are consistent with the clinical applicability demonstrated in Section 2.4.2, now extended to four fully independent proteins not involved in any stage of model development.

To illustrate the performance of our top-ranking models relative to some widely used predictors, we compared them with REVEL, AlphaMissense, SIFT, CADD and PrimateAI, methods commonly used as reference baselines in CAGI assessments, evaluated on the same ClinVar variant sets (Figure 8). The QAFI models ranked among the two best methods in all four proteins, and first in LPL (AUC 0.999) and TSC2 (0.922).

## 3. Discussion

Responding to the call to resolve the genotype-to-phenotype equation, a prerequisite for genomic medicine [1], we framed variant effect prediction as the modeling of a molecular endophenotype. This framing established two objectives for our work: first, to develop a method capable of accurately resolving this molecular layer; and second, to ask how far the resulting predictions carry into clinical classification. The QAFIsplit framework addresses the first objective. By reformulating prediction as a two-stage decomposition—first estimating the mutation tolerance of a position, and then modeling the specific deviation introduced by a substitution—QAFIsplit yields a biophysically grounded, mechanistically intuitive predictor. Crucially, this architectural choice does not sacrifice quantitative accuracy; as demonstrated by our benchmarks, it achieves state-of-the-art quantitative performance on independent functional assays, surpassing black-box alternatives in the vast majority of protein domains.

The success of this two-stage decomposition lies in its alignment with the biophysical reality of proteins. A variant’s functional impact is not generated in a vacuum; it is heavily conditioned by the local structural and energetic environment of the residue. Our results show that explicitly modeling this environment as “mutation tolerance” provides a highly informative prior for variant-level prediction, in accordance with Yu et al. [28]. The ability of the mtPSPs to recapitulate position-specific tolerance profiles—such as accurately identifying disordered regions in PRKN via AlphaFold pLDDT scores—confirms that our expanded feature set effectively captures the determinants of positional constraint. By separating this first-order effect from the second-order perturbation caused by a specific amino acid change, the framework reduces the burden on the downstream regressor, leading to more robust predictions.

This mechanistic grounding translates into tangible benchmark advantages. On the large-scale Beltrán et al. [26] dataset, QAFIsplit 3.0 outperformed AlphaMissense in 86% of protein domains and matched the performance of specialized, assay-tailored versions of protein language models like ESM-1v. We attribute this to the explicit inclusion of structurally informed descriptors—such as the normalized contact energy (NCE-NR) and evolutionary sequence context (neco features)—which capture local physicochemical constraints that deep learning representations, whether structure-based or LLM-derived, may implicitly encode but cannot explicitly leverage for out-of-distribution generalization. Notably, QAFIsplit 3.0 exhibited a “compensatory tail,” maintaining high accuracy on domains where other predictors failed. This suggests that the QAFI framework captures complementary aspects of variant biophysics, making it particularly valuable as a robust, stand-alone tool for newly characterized domains.

Translating quantitative functional scores into clinical utility presents a distinct challenge, as clinical genetics still relies heavily on binary pathogenic/benign classifications. Here the endophenotype route proves efficient, as postulated by Masica and Karchin [2]. Trained exclusively on continuous DMS data and never exposed to a clinical label, QAFIsplit 3.0 reached an AUC of 0.90—outperforming most tools in this clinical ecosystem, and sitting well above the median of that ecosystem, and close to the reference set by its own feature representation (0.92 for QAFIclas, trained directly on clinical labels). A part of what separates it from the upper end of that range (0.94–0.96) is the current accuracy of endophenotype prediction, and that part should narrow as this resolution improves. Another part, however, may not: clinical labels rest on evidence that lies outside the molecular layer—segregation and trio studies, clinical observation, and, for some tools, population allele frequencies—which a model confined to the endophenotype neither uses nor aims to. Consistent with this, adding QAFIsplit 3.0 to a meta-predictor of established tools (QAFIMeta) left their performance intact (0.94 to 0.95), showing that a continuous, biophysically grounded score can join the clinical toolkit without diluting it.

This behavior was further examined prospectively, under blind conditions, in the CAGI 7 community experiment. QAFI-based models—both the stand-alone predictor and meta-predictor strategies—were submitted to four independent challenges on proteins entirely absent from model development (ATP7B, LPL, ARSA, and TSC2), where their strong pre-submission validation on clinical variants (Section 2.4.3) was borne out in a fully blind setting. Communications from the organizers indicated that our models were among the better-performing entries, with formal rankings to appear in the CAGI 7 special collection. This extends the value of continuous, mechanistically grounded scores—alone or combined with existing classifiers—to genuinely unseen prediction settings.

An interesting technical observation emerges from the internal validation: while incorporating mutation tolerance as a feature (PSPsplit 1.0) consistently improved predictions, explicitly modeling the residual deviation from that positional value (PSPsplit 2.0) did not. We hypothesize that predicting residuals amplifies noise in the DMS median estimates, whereas feeding the predicted tolerance directly into XGBoost allows the non-linear model to learn the interaction between that positional estimate and the physicochemical properties of the new amino acid. This highlights an important balance in interpretable machine learning: explicit biological decomposition is powerful, but it must be coupled with algorithms that can handle the inherent noise of experimental biological data.

Despite these advances, limitations remain. The generalization gap observed between protein-specific predictors (PSPs) and the ensemble framework indicates that transferring local mutational landscapes across proteins is non-trivial. The failure to accurately predict SNCA (α-synuclein) underscores this: intrinsically disordered proteins violate the globular packing assumptions that underpin many of our structural features. Future iterations of QAFI could address these limitations by integrating disorder-specific predictors, post-translational modification annotations as positional priors, or graph neural networks capable of modeling long-range allosteric effects. Modeling protein complexes would be particularly valuable, so that residues at interaction surfaces are described in their bound rather than monomeric context.

In conclusion, the QAFI family of predictors demonstrates that high-resolution quantitative prediction and mechanistic interpretability are synergistic, not mutually exclusive. As the field moves beyond binary variant classification, frameworks like QAFIsplit will be essential for translating the full complexity of the genotype-to-phenotype map into actionable medical knowledge.

## 4. Materials and Methods

### 4.1. DMS Variant Datasets

We compiled 60 deep mutational scanning (DMS) datasets to train the protein-specific predictors (PSPs) that form the core of QAFI. Thirty were inherited from our previous work [21], and thirty were newly selected from the ProteinGym collection [29] using the following criteria: (i) the assay contained more than 1000 single missense variants; (ii) when several assays existed for the same protein, the one with broader positional coverage was kept; (iii) mutation coverage exceeded 50% of residue positions within the assayed region; (iv) an AlphaFold model was available [23,30]; and (v) a multiple sequence alignment (MSA) could be built. These criteria ensure sufficient statistical power for regression modeling and the availability of complementary structural and evolutionary features. The full protein list and metadata are given in Table S4.

### 4.2. Score Normalization

Raw DMS scores are assay-specific and not directly comparable across experiments. To integrate the 60 datasets into a common framework, we adapted the normalization strategy of Gray et al. [7], which was also used in its original form in QAFI 1.0 [21]. Scores were:

- Inverted where needed, so that higher scores consistently indicate higher functional capacity;
- Expressed as the log_2_ fold-change relative to the wild type;
- Rescaled so that wild-type-like variants take a value of 1 and the median of the bottom 1% of variants—a conservative proxy for complete loss of function [31]—takes a value of 0.

Scores outside this range were retained, as they carry biologically meaningful information: values above 1 correspond to gain of function, and values below 0 to variants more deleterious than the bottom 1% reference. Since that reference is the median of the bottom 1%, about half of those variants fall below zero by construction (0.51% of all scores). Normalized variant-level scores for all 60 datasets are provided in Table S5.

### 4.3. Functional Validation Dataset

QAFI models were validated in the large-scale mutagenesis dataset of Beltran et al. [26]. Entries without valid UniProt identifiers or AlphaFold structures were removed, retaining only missense variants and excluding proteins with residue-numbering mismatches between the UniProt sequence and the AlphaFold model. The final set contained 402 protein domains and 492,096 missense variants.

### 4.4. Dataset of Clinically Annotated Variants

We assembled a set of clinically annotated missense variants from the January 2025 releases of ClinVar [32] and Humsavar [33]. Variants labeled Pathogenic, Likely pathogenic, or Pathogenic/Likely pathogenic were grouped as pathogenic, and those labeled Benign, Likely benign, or Benign/Likely benign as benign. We retained only variants with a review status of at least one star, excluding variants of uncertain significance and those with conflicting classifications. Variants deposited before 2021 were excluded to minimize overlap with the training sets of the predictors evaluated here. All proteins present in the DMS training set were excluded to avoid circularity. The resulting set comprised 42,173 variants across 2012 human proteins (13,167 pathogenic, 29,006 benign).

This collection is used here to compare predictor performance. Because coverage differs across methods, all cross-method comparisons were carried out on the subset scored by every one of them: 12,787 variants (6606 pathogenic, 6181 benign).

### 4.5. Protein Structure Dataset

To parameterize the structure-based features, we curated a non-redundant set of high-resolution structures from PISCES [34] (X-ray structures at ≤50% sequence identity, resolution ≤ 1.8 Å, R-factor ≤ 0.25). For each protein we kept only PDB chains with a one-to-one UniProt match and 100% sequence identity after alignment, using chain A when multiple chains were present. Structures were processed with ARPEGGIO version 1.4.4 [35] to compute atomic contacts, and entries with duplicate residue indices or insufficient MSA depth were excluded. After filtering, 4355 structures with valid contact annotations were retained.

### 4.6. Multiple Sequence Alignments

MSAs were built following Riera et al. [36]. For each target protein, homologs were retrieved from UniRef100 [37] with PsiBlast (BLAST+ version 2.16.0) [38] (E-value 0.001, two iterations); sequences with less than 40% identity to the target were discarded; and the remaining sequences, together with the query, were aligned with Muscle version 3.8.1551 [39].

### 4.7. Feature Representation

Each variant was represented by sequence- and structure-based features. Fourteen features were taken from our previous work [21], spanning evolutionary, physicochemical, and structural information (e.g., Blosum62 scores, Shannon entropy, PSSM, pLDDT, solvent accessibility, and Miyazawa–Jernigan contact-energy terms). In this study we extended the representation with 14 new descriptors of the native residue and its environment, parameterized from the curated set of 4355 structures. They are organized into four conceptual groups—an energy-based descriptor (NCE-NR), sequence-context descriptors (the neco family), probability-density descriptors (PDFFs), and mutation tolerance—described below. Full definitions for every feature are listed in Table S6.

### 4.8. Normalized Contact Energy of the Native Residue (NCE-NR)

NCE-NR is a coarse-grained measure of the contact energy of the wild-type residue within its structural environment. Using the Miyazawa–Jernigan (MJ) potential [40], we identify the spatial neighbors of the native residue (side-chain centroids within 6.5 Å, or Cα for glycine) and sum their MJ interaction energies to obtain $E_{\mathrm{native}}$. This is normalized by the maximum contact energy observed for that amino acid type across the curated dataset,

(1) $$\mathrm{NCE}\text{-}\mathrm{NR}=\frac{E_{\mathrm{native}}}{E_{\mathrm{native}\text{\_}\max}}$$

which removes intrinsic contact biases across amino acid types and yields a relative measure of local structural stability.

### 4.9. Evolutionary Compatibility with Neighbor Sequence Context (neco)

To capture context-dependent substitution effects, we introduced the neco family of features, which quantify how well a substitution fits its surrounding sequence environment. Unlike position-wise conservation scores, neco features model dependencies between the target residue and conserved neighboring residues using log-odds scores derived from MSAs. We defined four variants—neco1, neco2, neco3, and neco12—representing sequence context at different distances.

The immediate-neighbor score, neco1, evaluates compatibility with the first neighbors at positions i−1 and i+1. For a native residue $A_{i}$ flanked by $X_{1}$ and $Y_{1}$ and substituted by B,

(2) $$\mathrm{neco}1=\ln\left(\frac{P(X_{1}\;B\;Y_{1}\;|\;\mathrm{human}=X_{1}A_{i}Y_{1})}{P(X_{1}\;*\;Y_{1}\;|\;\mathrm{human}=X_{1}A_{i}Y_{1})}\right)$$

where the numerator is the probability of observing the mutant residue B under the conserved flanking context and the denominator (∗ = any central residue) reflects the background frequency of that context in the MSA. The remaining variants follow the same log-odds form but use more distant or combined neighbors: neco2 and neco3 use the second (i±2) and third (i±3) neighbors, respectively, and neco12 jointly uses the first and second neighbors on both sides (i±1,i±2). Each neco feature is a 20 × 20 matrix in which higher values indicate substitutions more compatible with the local evolutionary context; their explicit forms are given in Table S7.

### 4.10. Probability Density Function Features (PDFFs)

Building on previously defined residue-level descriptors (e.g., pLDDT, contact energy, conservation entropy), PDFFs quantify how the empirical distribution of a feature shifts when a native residue A is replaced by W, relative to the background at all positions carrying A. For a substitution A→W and a feature value x,

(3) $$r(x)=\ln\left(\frac{p(x\;|\;A\to W)}{p(x\;|\;A)}\right)$$

where p(x | A→W) is the density of the feature among homologs in which A is replaced by W and p(x | A) is the background density at all positions carrying A, regardless of mutation status. Because empirical densities can be noisy in rare substitution contexts, the numerator and denominator distributions were each fitted with three-component Gaussian mixture models (and, for a small number of features with atypical distributions, alternative functional forms) before computing r(x). The final PDFF of each feature is a 20 × 20 matrix of scoring functions r(x) that can be applied to any substitution in any protein.

### 4.11. Mutation Tolerance

To capture the overall sensitivity of a residue position to mutational effects, we explicitly model mutation tolerance. It is operationally defined as the median DMS score observed at each position; constrained positions show low mutation tolerance, whereas permissive positions show high mutation tolerance.

Unlike the other descriptors, mutation tolerance cannot be computed directly for a new position but must itself be predicted from local features. For each protein, the per-position median DMS score is paired with a vector of ten position-level features $x_{p}$, three of them derived from the multiple sequence alignment (PSSM, Shannon entropy, and entropy of sequence neighbors) and seven computed from the protein’s AlphaFold model (pLDDT, pLDDT bin, Colasi, fraction of conserved 3D neighbors, FANC, FBNC, and NCE-NR), and a multiple linear regression (MLR) is fitted across positions

(4) $${\tilde{y}}_{p}=\beta_{0}+x_{p}^{⊤}\beta +\varepsilon_{p}$$

where $\beta_{0}$ is the intercept, β the regression coefficients, and $\varepsilon_{p}$ the error term. Each model thus has eleven fitted parameters, and is fitted on as many observations as there are positions covered by the assay (median 302, range 54–934; Table S4). The fitted model predicts the mutation tolerance at any position from its feature vector; this value is the input used by the split models below.

The AlphaFold-based features were computed using structures retrieved from the AlphaFold Protein Structure Database [30], v4 release, using the single top-ranked model. The AFDB has since been updated (to v6); to ensure reproducibility, the exact v4 structures used in this study are deposited in our GitHub repository “https://github.com/shaopeiye0-oss/QAFI2 (accessed on 16 August 2026)”. All the predictive performances listed have been obtained using v4 structures. Because the predicted tolerance feeds the second stage, we verified that this choice does not affect our results: mtPSP predictions recomputed from v6 structures are essentially identical to the v4-based ones (r ranging from 0.963 to 1.000, median 0.999; Figure S13).

### 4.12. Feature Standardization

All features were standardized to zero mean and unit variance using *z*-score standardization,

(5) $$x^{\prime }=\frac{x-\mu_{\mathrm{train}}}{\sigma_{\mathrm{train}}}$$

where $\mu_{\mathrm{train}}$ and $\sigma_{\mathrm{train}}$ are computed on the training set only and then frozen and reused for the validation and test data, preventing information leakage from the held-out sets.

### 4.13. Baseline Variant Effect Models: PSP 1.0 and PSP 2.0

To quantify the improvements introduced by the two-stage design, we defined two baselines at the protein-specific (PSP) level. PSP 1.0 corresponds to the original QAFI protein-specific predictor [21], a separate multiple-linear-regression model for each of 30 proteins using 14 sequence- and structure-based features. PSP 2.0 is an updated version that extends both the feature set and the number of proteins—a separate model for each of the full set of 60 proteins, using the 28 features described above—and replaces multiple linear regression with XGBoost regression. Both predict variant effects directly from the full feature set, without explicitly separating position-level from mutation-specific effects.

### 4.14. Two-Step Position-Informed Regression

Because the functional impact of a mutation is strongly shaped by the structural and evolutionary constraints of its position, we developed a two-step regression framework that separates position-level effects from mutation-specific deviations. In both variants, the first step estimates the mutation tolerance ${\tilde{y}}_{p}$ at each position as described above; the second step uses this estimate, together with the mutation-level feature vector $x_{i}$, to predict the variant effect.

In PSPsplit 1.0, the predicted mutation tolerance is added as an extra input feature to an XGBoost regressor,

(6) $${\hat{y}}_{i}=f_{\mathrm{XGB}}([x_{i},\;{\tilde{y}}_{p}])$$

In PSPsplit 2.0, the model instead predicts the residual between the observed score and the predicted tolerance,

(7) $$r_{i}=y_{i}-{\tilde{y}}_{p},\;\;{\hat{r}}_{i}=f_{\mathrm{XGB}}([x_{i},\;{\tilde{y}}_{p}]),\;\;{\hat{y}}_{i}={\tilde{y}}_{p}+{\hat{r}}_{i}$$

where $y_{i}$ is the observed (normalized) DMS score of variant i, $r_{i}$ the residual relative to the predicted tolerance ${\tilde{y}}_{p}$, ${\hat{r}}_{i}$ the residual predicted by the XGBoost regressor $f_{\mathrm{XGB}}$ from the mutation-level features $x_{i}$, and ${\hat{y}}_{i}$ the final predicted variant effect. This formulation isolates the mutation-specific perturbation from the predicted tolerance. Both frameworks were evaluated by leave-one-position-out cross-validation within each protein [41], with performance assessed by Pearson (r) and Spearman (ρ) correlation, mean absolute error, and root mean squared error. All models were implemented with the XGBoost version 2.1.4 in scikit-learn version 1.6.1 [42], using hyperparameters fixed across proteins.

### 4.15. Ensemble Architecture and Aggregation

The protein-specific predictors (PSPs) are the building blocks of the general QAFI models: a variant is scored by aggregating predictions from many PSPs, so that any variant in any protein can be predicted even when no DMS data exist for that protein. All QAFIsplit variants share the same three-step pipeline:

Step 1—Training. A PSP is trained independently on each of the 60 proteins.

Step 2—Selection. A fixed subset of *N* = 30 PSPs—those generalizing best across proteins—is selected using the nested leave-one-protein-out procedure described below (Ensemble construction and size selection).

Step 3—Aggregation. The 30 selected PSPs are combined into a single prediction. The choice of PSP type and fusion rule defines the QAFIsplit versions, where ${\hat{y}}_{i,m}$ denotes the prediction of model m for variant i.

QAFIsplit 1.0 and QAFIsplit 2.0 (median fusion). These aggregate the top 30 PSPsplit 1.0 and PSPsplit 2.0 models, respectively, giving every model equal influence through the median,

(8) $${\hat{y}}_{i}^{\mathrm{median}}={\mathrm{median}}_{m=1,\ldots ,30}\;{\hat{y}}_{i,m}$$

This is robust but treats all 30 models as equally informative, ignoring that they differ in how reliable their predictions are.

QAFIsplit 3.0 (correlation-weighted fusion). This version uses the same 30 PSPsplit 2.0 candidates as QAFIsplit 2.0 but replaces the median with a weighted average that favors models agreeing with the ensemble consensus. Each candidate is applied to the validation proteins, and the pairwise Pearson correlations among the 30 predictions are computed; the reliability of model m is its mean correlation with the other 29 candidates,

(9) $${\bar{r}}_{m}=\frac{1}{29}\sum_{j\neq m}C_{mj}$$

where C is the correlation matrix. The reliabilities are normalized into weights and used to combine predictions as follows:

(10) $$w_{m}=\frac{{\bar{r}}_{m}}{\sum_{j=1}^{30}{\bar{r}}_{j}},\;\;{\hat{y}}_{i}^{\mathrm{fusion}}=\sum_{m=1}^{30}w_{m}\;{\hat{y}}_{i,m}$$

Candidates systematically out of step with the others are thus down-weighted, while consistent models contribute more strongly.

Baseline ensemble models. Two ensemble baselines frame these results. QAFI 2.0 applies the same pipeline to the PSP 2.0 predictors (top-30 selection, median aggregation), so that its gain over QAFI 1.0 reflects the expanded features and XGBoost regression alone, not the two-stage split. QAFI 1.0 is the published ensemble [21]—the median of its ten original PSP 1.0 predictors.

### 4.16. Size and Composition of QAFI Ensemble Models

A key feature of the QAFI framework is its ensemble strategy, in which predictions from many protein-specific models are aggregated into a single, generalizable variant-effect score. Two choices define the ensemble: its size (the number of PSPs, N) and the identity of its members. Both were determined with a nested leave-one-protein-out procedure (Figure S7A).

In the outer loop, each protein *k* is held out in turn (*k* = 1…60) and its PSP excluded. In the inner loop, each of the remaining 59 PSPs, *i*, is applied to the DMS data of the other 58 proteins (excluding both *k* and *i*’s own protein), yielding 58 Pearson correlations whose median is taken as the performance of PSP *i*. The 59 PSPs are then ranked by this median, and the top N are recorded. Repeating the outer loop over all 60 proteins yields 60 such ranked lists, and the N PSPs appearing most frequently across them define the deployed ensemble.

The same procedure provides an unbiased performance estimate: for each held-out protein *k*, the N PSPs selected from the others are applied as an ensemble to its DMS data, and the Pearson correlation between predicted and observed scores is computed. This yields 60 cross-validated correlations across the full dataset. The ensemble size was chosen by repeating the whole procedure for *N* = 10, 15, 30, 45, and 60 and, for each protein, identifying the N with the highest correlation; the value winning in most proteins, *N* = 30, was selected (Figure S7B,C).

### 4.17. Clinical Variant Classifier (QAFIclas)

As a reference for the classification performance attainable from our feature representation, we trained QAFIclas, an XGBoost classifier that uses the same features as QAFIsplit 3.0 but is trained directly on clinical labels rather than on continuous DMS scores. QAFIclas is intended only to indicate how much clinically relevant signal these features can capture when optimized for classification; it is not proposed as a variant classifier for practical use.

QAFIclas was fitted on the curated pathogenic/benign set described above and evaluated on the same common set of 12,787 variants used throughout Section 2.4.2, reaching an AUC of 0.92. The model used reg_alpha = 10, reg_lambda = 10, max_depth = 4, learning_rate = 0.1, subsample = 0.9, colsample_bytree = 0.8, n_estimators = 200, and log-loss as the evaluation metric; its output ranges from 0 (pathogenic) to 1 (benign).

### 4.18. Functional Benchmarking

QAFI was benchmarked against existing variant effect predictors on the Beltran et al. dataset [26] described above. The dataset provides scores from twelve representative predictors—ESM1v_domain [11], RaSP [43], ddMut [44], FoldX [45], popEVE [46], EVE [9], Tranception [47], EVE_domain [9], rsASA (this is a residue accessible surface area computation), ThermoMPNN [48], AlphaMissense [12], and ESM1v_full-length [11]—used for comparison in quantitative functional prediction.

While the predictors above were all evaluated under identical conditions, ThermoMPNN [48] required a separate treatment because it was trained on the mega-scale dataset of Tsuboyama et al. [49], which overlaps substantially with the Beltrán et al. benchmark [26]. To avoid the data leakage that would occur if it were evaluated on domains from its training set, we restricted its comparison to the homolog-free subset defined by Beltrán et al. [26]—benchmark domains with no Pfam homology to the Tsuboyama et al. training set. All other predictors were compared on the full 402-protein domain benchmark.

### 4.19. Circular Projection Plots

Circular projection plots are a representation aimed at showing, for a given sample, how two methods outperform each other to help detect trends in the data. This is particularly useful when the differences between methods are small and it is hard to tell whether one of them is systematically predominant. In our case the performance parameter is the Pearson correlation coefficient, and the sample is the set of 402 protein domains from Beltrán et al.’s dataset on which the methods are validated. The procedure works as follows:

- For each domain, the Pearson correlations achieved by the two methods, rA and rB, define a point in a two-dimensional plane. The angle of the vector from the origin to that point is obtained as θ = arctan 2(rB, rA), and then rotated by 45° to help visual interpretation. Normalizing the vector places the resulting point on a ring of radius 1: if it falls to the right of the y-axis, rA > rB and method A outperforms method B; if it falls to the left, the opposite holds. Note that the plot conveys the sign of the difference between methods, not its magnitude;
- Applying this procedure to all the domains in the sample results in a distribution of points along the ring, corresponding to the different comparisons;
- To facilitate the visual analysis, the distribution of points is rendered as a heatmap in which darker tones correspond to higher concentrations of points. A needle indicates the direction defined by the pair of median correlations.

Since both correlations are typically positive, all angles fall in the upper semicircle. The percentage shown inside each semicircle is the fraction of domains where the first method performs better, and its statistical significance is assessed by a one-sided exact binomial test. In this work, circular projection plots are used to compare QAFIsplit 3.0 with external predictors (Figure 5) and with the other members of the QAFI family (Figure S10).

### 4.20. Residual Deviation Analysis

As a scale-sensitive complement to the Pearson correlation, we computed, for each of the 402 domains, the root mean squared error between predicted and observed normalized DMS scores.

This comparison was carried out at two levels. First, it was performed on the raw outputs of each method, as returned by its authors. Most of the methods compared here return quantities on scales unrelated to the normalized functional scale—relative solvent accessibility, folding free energies in kcal/mol, or sequence log-likelihoods—so their deviation reflects primarily this difference in units rather than predictive error. Second, it was carried out after min–max-rescaling each method to [0, 1] within each domain, a transformation that is not part of the original methods and that we applied to make the comparison possible on a common scale. The experimental scores were left unmodified in both cases, since reproducing the normalized DMS scale is precisely the task being evaluated. Methods whose output increases with loss of function (AlphaMissense, EVE, FoldX, RaSP and thermoMPNN) were inverted beforehand, so that in all cases higher values correspond to higher fitness.

### 4.21. Clinical Benchmarking

QAFIsplit 3.0 was benchmarked against a panel of established variant effect predictors on the curated pathogenic/benign set described above. Most predictor scores were retrieved through VEP (release 113) [50], which at the time of analysis drew on the dbNSFP v4.7c plugin [51]; scores for popEVE and CPT-1, which are not available through VEP, were obtained directly from their respective official sources (accessed on 30 September 2025). All predictors were evaluated on the common subset of variants scored by every method (*n* = 12,787), and scores were oriented, where necessary, so that higher values consistently indicate greater predicted pathogenicity. Discriminative performance was quantified as the area under the ROC curve (AUC).

For interpretation, the predictors were organized into three reference groups. (1) Tools developed for clinical variant interpretation—several of which have been specifically calibrated for ACMG/AMP guideline evidence [18]—is the first group. To provide a comprehensive view of the clinical ecosystem, this group includes a representative set of such classifiers available in the dbNSFP v4.7c plugin (e.g., SIFT [52], PolyPhen [53], CADD [54], GERP++ [55], PhyloP [56], FATHMM [57], MPC [58], PrimateAI [59], MutPred2 [60], REVEL [15], VEST4 [61], ClinPred [62], and BayesDel [16]). (2) The second group is QAFIclas, trained directly on clinical labels as a theoretical reference for the performance attainable from the feature representation (see above). (3) The third is a group of advanced machine learning methods not trained on clinical labels (e.g., AlphaMissense [12], EVE [9], CPT-1 [63], popEVE [46]). We further assessed whether QAFIsplit 3.0 contributes complementary signal by combining it with AlphaMissense, popEVE, CPT-1, REVEL and BayesDel through median aggregation (QAFIMeta), evaluated on the same common subset.

### 4.22. Writing Support

The text of this article was reviewed for grammar and clarity using an AI language model. All suggested corrections were carefully checked to ensure they faithfully reflect our intended meaning and results.

## Figures and Tables

**Figure 1:**
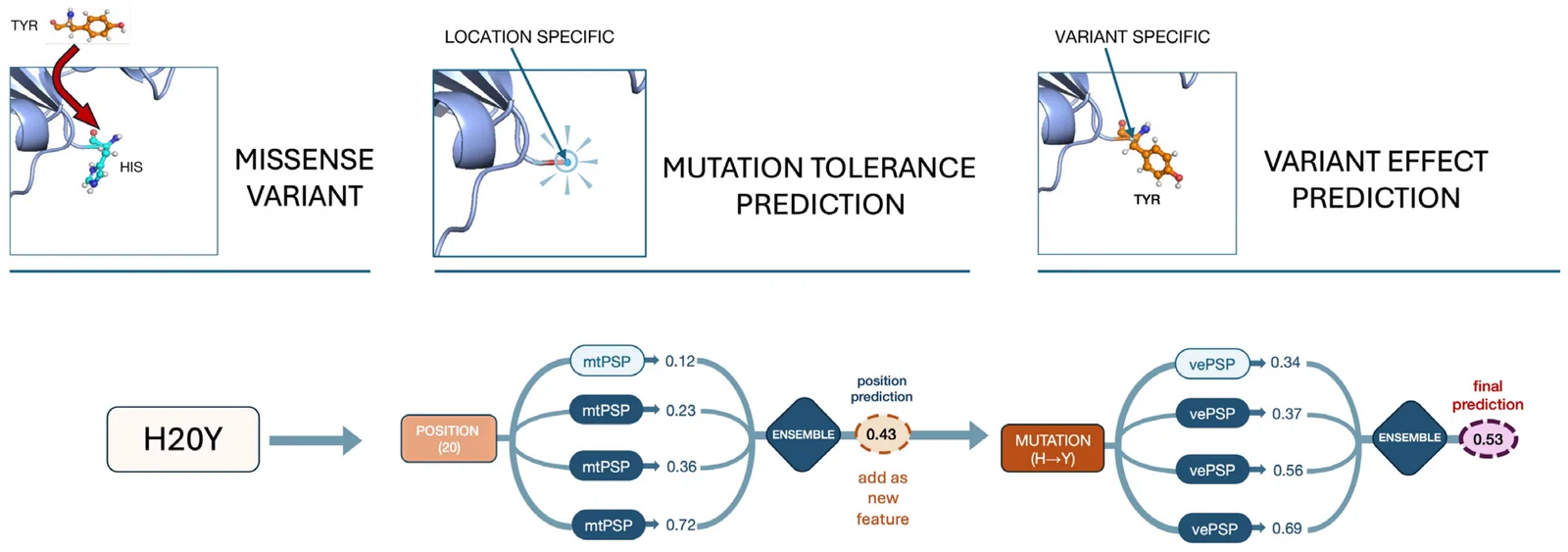
**Overview of the QAFIsplit two-stage ensemble framework.** Top icons (left to right) denote the three sequential stages of the pipeline: input missense variants, mutation tolerance prediction, and ensemble-based variant effect prediction, each represented by a schematic protein structure. The central workflow diagram illustrates the QAFIsplit prediction pipeline. A missense variant (input box) is passed to the mutation tolerance module, which generates a continuous positional score (mutation tolerance) reflecting the intrinsic sensitivity to mutations of that specific residue position, based on its local structural and evolutionary environment. This positional score is then incorporated as an additional feature into an ensemble model that aggregates multiple variant-level physicochemical properties to yield the final, variant-specific functional effect prediction.

**Figure 2:**
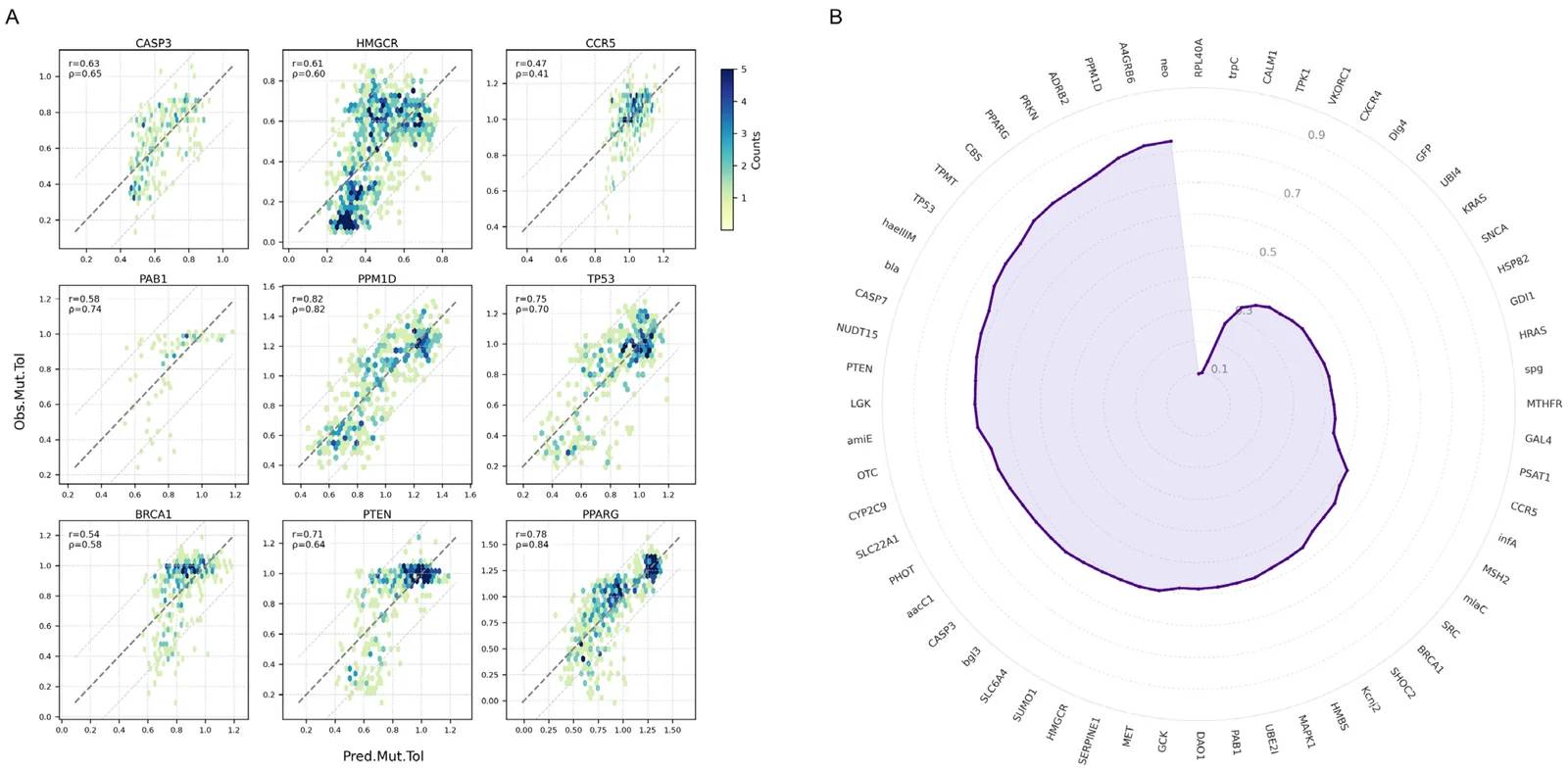
**Cross-validated performance of protein-specific mutation tolerance predictors (mtPSPs) across 60 proteins.** (**A**) Observed versus predicted mutation tolerance for nine representative proteins (CASP3, HMGCR, CCR5, PAB1, PPM1D, TP53, BRCA1, PTEN, and PPARG). Each panel displays the density of variant-level data points (color scale, counts per bin), with the dashed diagonal indicating perfect agreement. Pearson (r) and Spearman (ρ) correlation coefficients are shown for each protein. Both observed and predicted mutation tolerance can take slightly negative values at highly intolerant positions, since the zero of the normalized scale is set at the median of the most damaging variants (see Section 4.2). (**B**) Radial plot of per-protein Pearson correlations between predicted and observed mutation tolerance across all 60 proteins. Concentric rings indicate correlation values from 0.1 (inner) to 0.9 (outer). Protein names are arranged along the perimeter; the filled area reflects each protein’s predictive accuracy.

**Figure 3:**
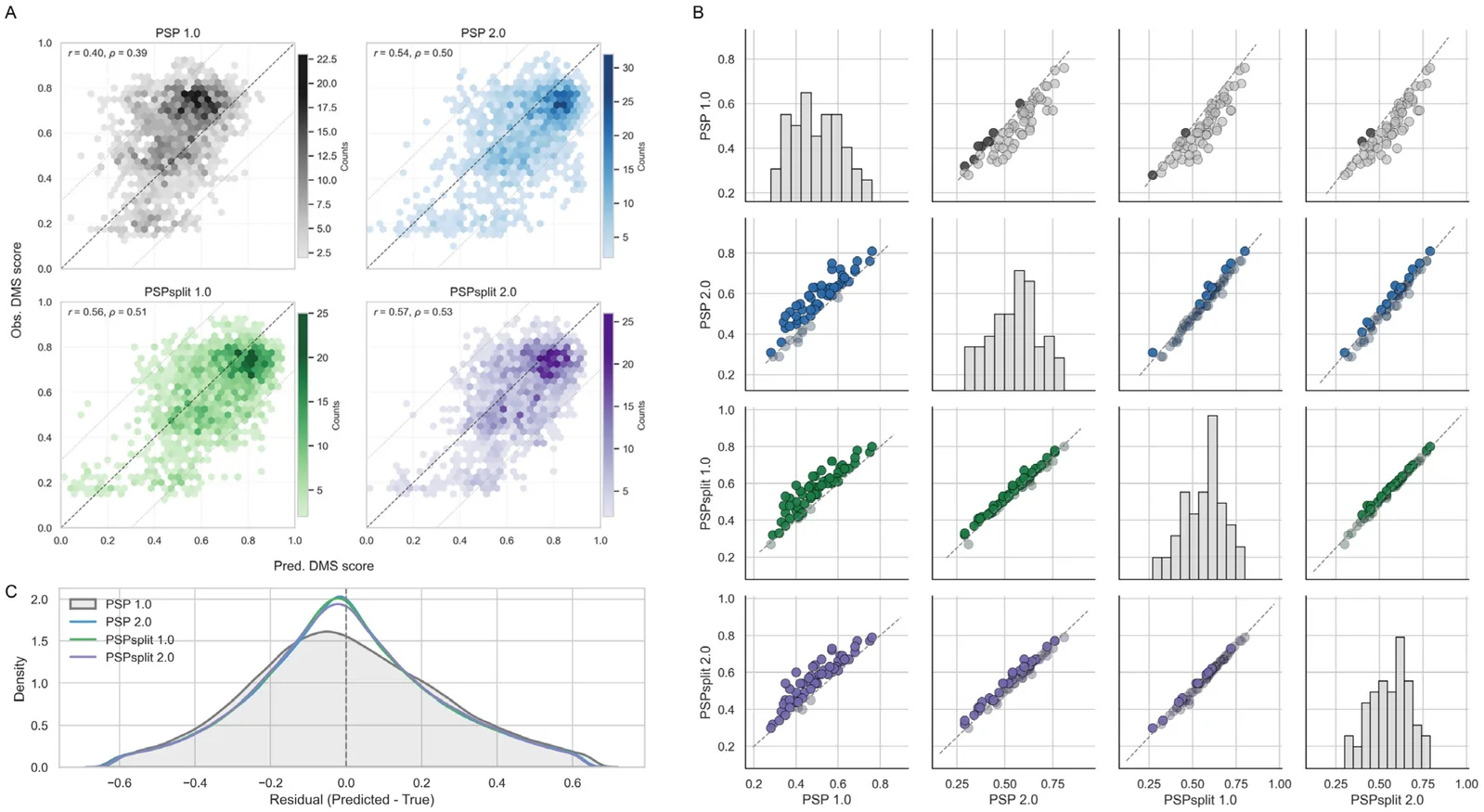
**Cross-validated variant-level prediction performance of vePSP models.** (**A**) Observed versus predicted DMS functional scores for VKORC1, shown as density plots for each of the four vePSP models: PSP 1.0 (gray), PSP 2.0 (blue), PSPsplit 1.0 (green), and PSPsplit 2.0 (purple). Color intensity reflects local point density (counts per bin). The dashed diagonal indicates perfect agreement; Pearson (r) and Spearman (ρ) correlation coefficients are shown for each model. (**B**) Pairwise scatter plot matrix of per-protein Pearson correlations across the four models. Each point represents one of the 60 proteins. Diagonal panels show the distribution of per-protein Pearson correlations for each model. Off-diagonal panels show pairwise comparisons between models, with the dashed diagonal indicating equal performance; points are colored according to the model on the corresponding row. (**C**) Density distribution of prediction residuals (Predicted−True) across all 60 proteins for each model (color scheme as in (**A**)). The dashed vertical line marks zero error.

**Figure 4:**
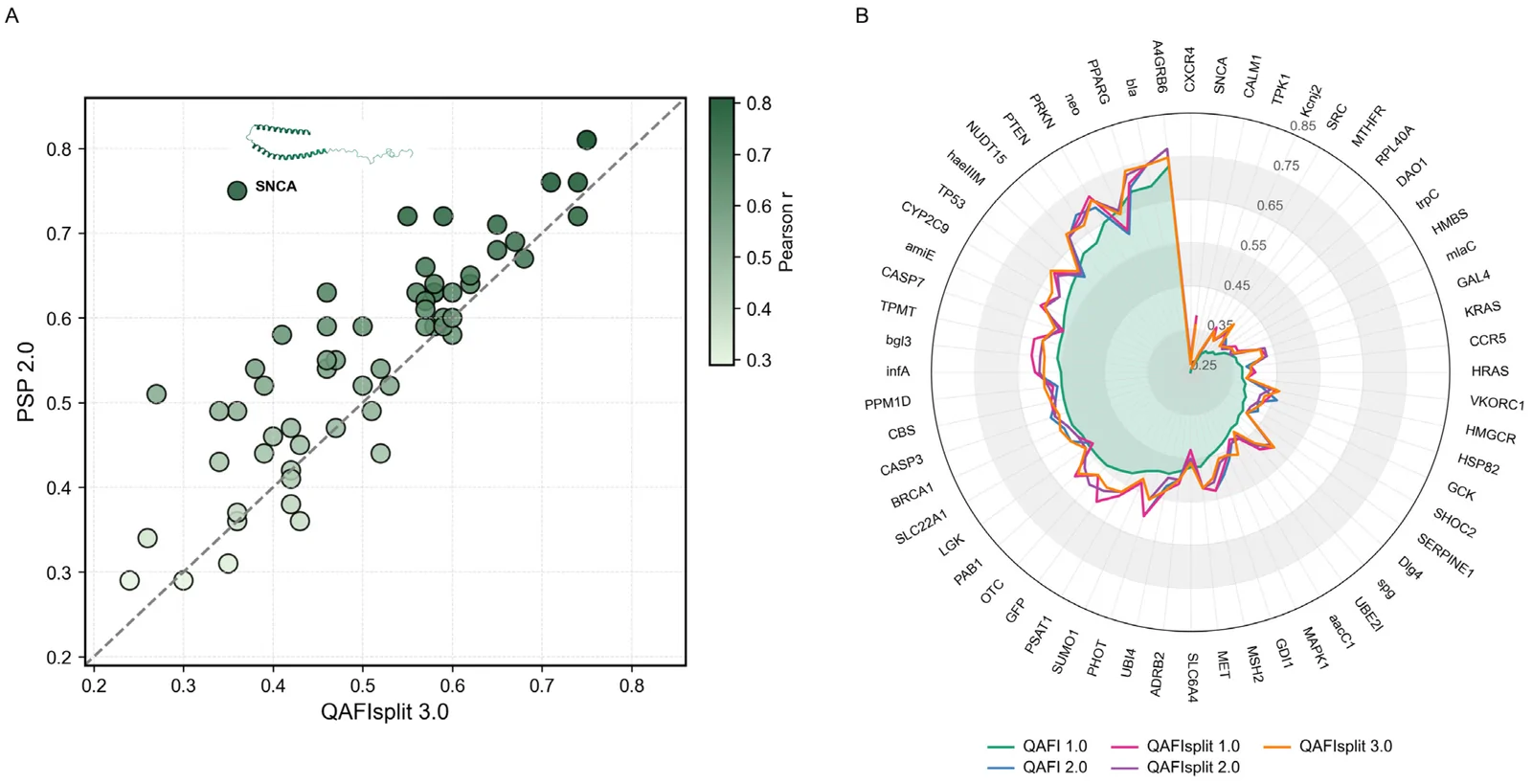
**Cross-validated performance of QAFI ensemble models.** (**A**) Per-protein Pearson correlations of PSP 2.0 (within-protein, y-axis) versus QAFIsplit 3.0 (cross-protein ensemble, x-axis) across the 60 proteins. Each point represents one protein, colored by its PSP 2.0 Pearson correlation (color scale, right). The dashed diagonal indicates equal performance; points above the diagonal reflect the within-protein advantage of PSP-level models over the ensemble. SNCA (α-synuclein) is highlighted as a consistent outlier; the inset shows its three-dimensional structure, illustrating its intrinsically disordered character, which likely accounts for its poor generalization under the ensemble framework. (**B**) Radial plot of per-protein Pearson correlations for all five QAFI ensemble models across the 60 proteins. Protein names are arranged along the perimeter; concentric rings indicate correlation values from 0.25 (inner) to 0.85 (outer). The shaded area corresponds to QAFI 1.0 (baseline, green); the other lines represent QAFI 2.0 (blue), QAFIsplit 1.0 (pink), QAFIsplit 2.0 (purple), and QAFIsplit 3.0 (orange).

**Figure 5:**
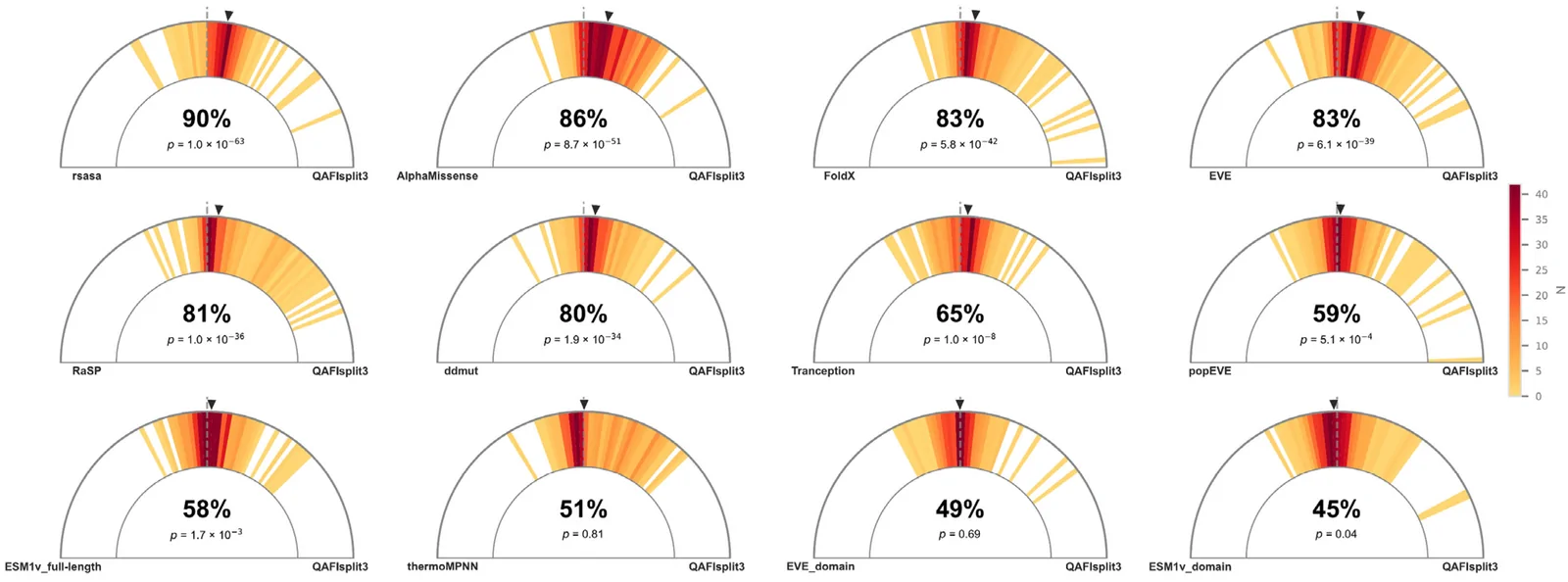
**Large-scale benchmarking of QAFIsplit 3.0 against state-of-the-art variant effect predictors across 402 protein domains.** Each panel compares QAFIsplit 3.0 against one alternative method. For each protein domain, the relative performance of the two methods—measured as Pearson correlation with experimental data—is represented as an angular position along the outer ring of the semicircle. For each comparison, per-domain correlations were computed on the variants for which both methods provide predictions. Domains where QAFIsplit 3.0 outperforms the competitor fall to the right of the vertical dashed line (equal performance); domains where the competitor outperforms QAFIsplit 3.0 fall to the left. The color of the outer ring reflects the local density of domains (yellow: low density; dark red: high density; scale N, right). The arrowhead indicates the overall tendency across all compared domains, based on median per-domain correlations. The percentage shown inside each semicircle indicates the fraction of domains where QAFIsplit 3.0 outperforms the respective competitor. Methods are ordered by this percentage in descending order (top left to bottom right). *p*-values are from one-sided exact binomial tests against the null hypothesis that both methods have equal probability of outperforming each other. See Section 4.19 for details on how these plots are built.

**Figure 6:**
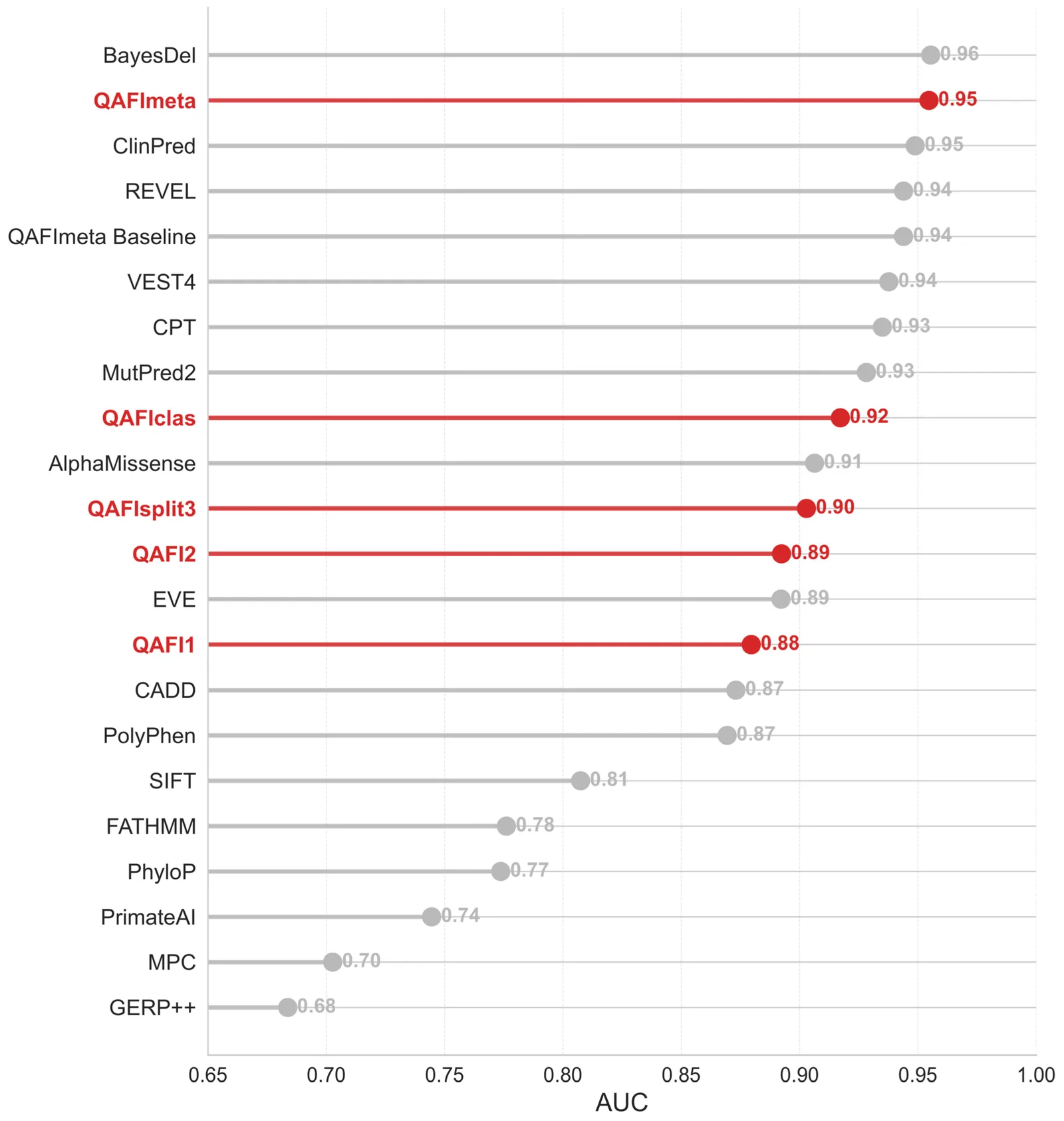
**Clinical variant classification performance of QAFIsplit 3.0 and comparator predictors.** AUC values for 12,787 clinically annotated variants (pathogenic/benign) across a panel of variant effect predictors. QAFI family methods (red) are shown alongside established clinical tools and advanced machine learning predictors (gray), ordered by AUC in descending order. QAFIclas, trained on clinical labels using the same 28 features as QAFIsplit 3.0, approximates the upper performance bound achievable by this feature space on the clinical classification task. QAFIMeta Baseline denotes the meta-predictor combining AlphaMissense, popEVE, CPT-1, REVEL, and BayesDel; QAFIMeta denotes the same combination with QAFIsplit 3.0 included.

**Figure 7:**
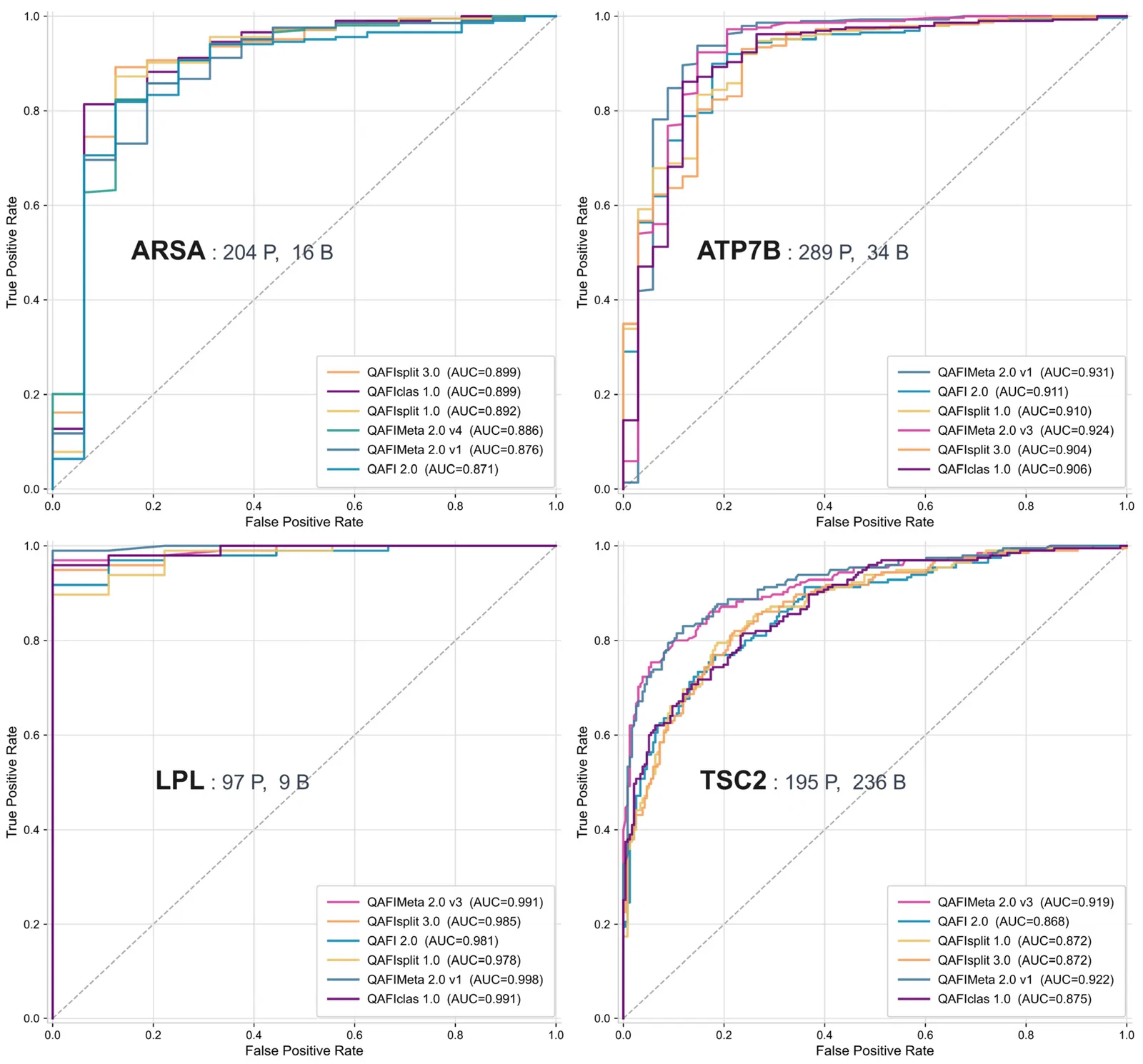
**Pre-submission clinical validation of QAFI models for the four CAGI 7 challenge proteins.** ROC curves for the six QAFI family models submitted to each challenge, evaluated on ClinVar-annotated pathogenic and benign missense variants for ARSA (204 pathogenic, 16 benign), ATP7B (289 pathogenic, 34 benign), LPL (97 pathogenic, 9 benign), and TSC2 (195 pathogenic, 236 benign). These evaluations were performed prior to submission and without access to challenge outcomes, serving as the primary evidence base for model selection. AUC values are shown in each legend.

**Figure 8:**
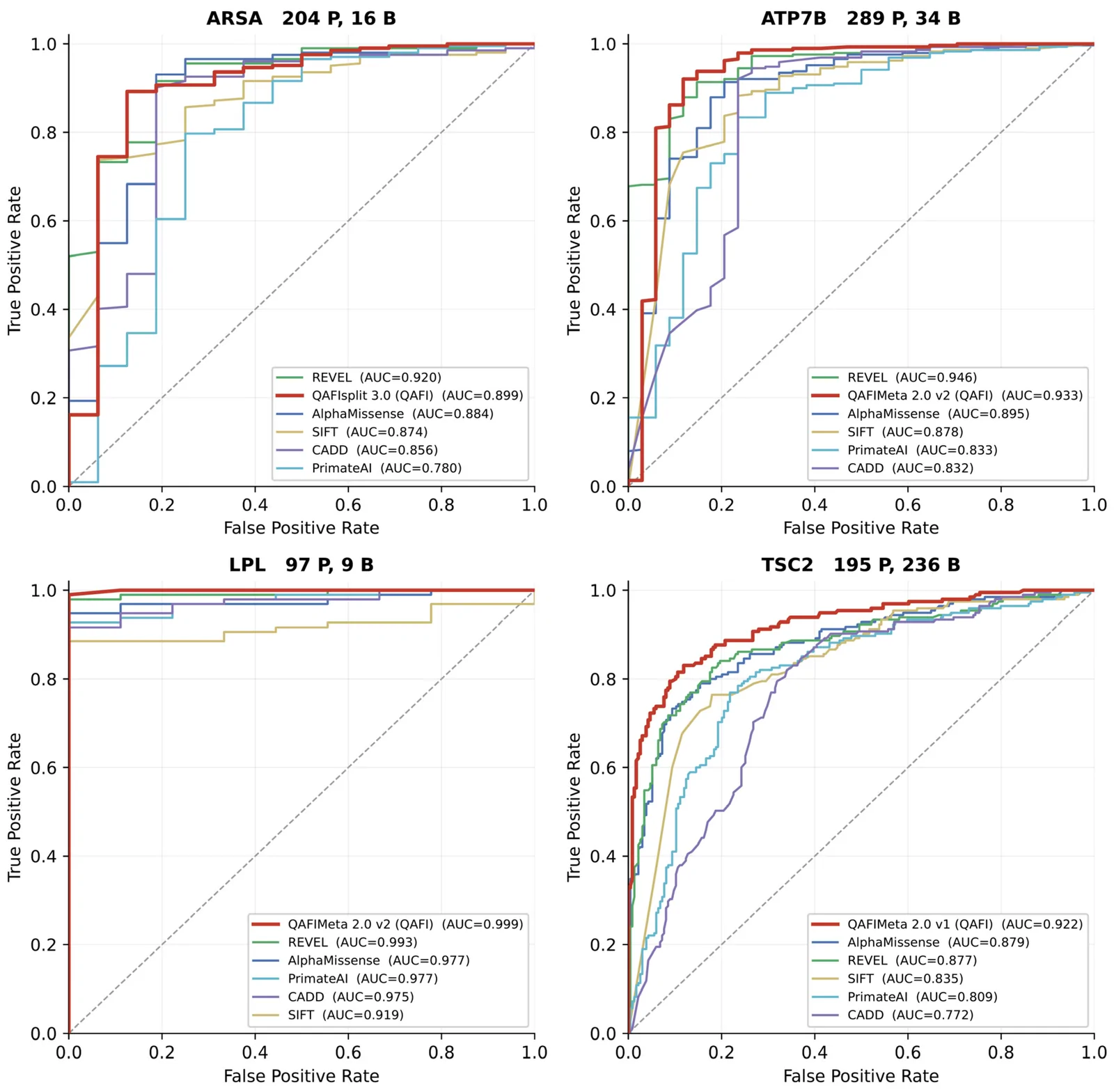
**Comparison of the top-ranking QAFI model for each CAGI 7 challenge protein with widely used variant effect predictors.** ROC curves for the best-performing QAFI model (red) and for REVEL, AlphaMissense, SIFT, CADD and PrimateAI, evaluated on the same ClinVar-annotated pathogenic and benign missense variants used in Figure 7: ARSA (204 pathogenic, 16 benign), ATP7B (289, 34), LPL (97, 9) and TSC2 (195, 236). The number of variants of each class is indicated in each panel heading. AUC values are shown in each legend; the dashed diagonal indicates random discrimination.

## Data Availability

The data underlying the figures and results of this study are provided in the Supplementary Tables. The large-scale mutational dataset of Beltrán et al. is available from its original source; derived data are available from the lead contact (S.Y.) upon request. All original code is publicly available at “https://github.com/shaopeiye0-oss/QAFI2 (accessed on 16 August 2026)” as of the date of publication. Any additional information required to reanalyze the data reported in this paper is available from the lead contact (S.Y.) upon request.

## Supplementary Materials

The following supporting information can be downloaded at: https://www.mdpi.com/article/10.3390/ijms27198777/s1.
