# Supplementary material for: Toward Advanced Quantitative Prediction of Missense Variant Effects
Source: Int J Mol Sci. 2026 Sep 30;27(19):8777. doi: 10.3390/ijms27198777 (PMC13664852; doi:10.3390/ijms27198777)
Supplement: Supplementary file 1 [file ijms-27-08777-s001.zip › SUPPLEMENTARY MATERIAL.pdf]

SUPPLEMENTARY MATERIAL (FIGURES AND TABLES)

SUPPLEMENTARY FIGURES

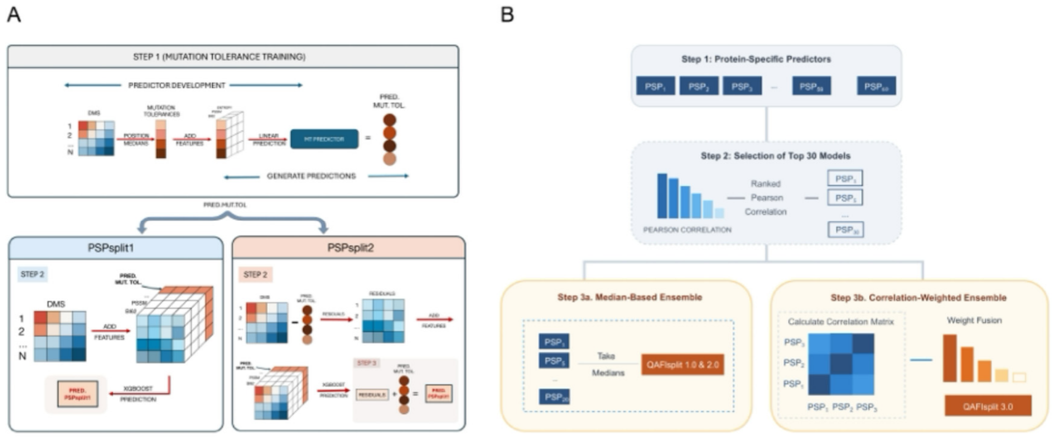

Figure S1. Detailed architecture of the QAFIsplit framework.

(A) Step-by-step workflow of the two-stage prediction pipeline. In Step 1, positional mutation tolerance is computed as the median functional effect across DMS data and used to train a linear mutation tolerance predictor (MT predictor). The resulting predicted mutation tolerances (Pred.Mut.Tol) feed into two alternative variant-effect prediction strategies: PSPsplit 1.0, which incorporates predicted mutation tolerance directly as an additional feature into an XGBoost model; and PSPsplit 2.0, which first computes residuals between observed DMS scores and predicted mutation tolerance, then predicts those residuals before reconstructing the final score. (B) Ensemble construction workflow. Step 1 pools all 60 protein-specific predictors (PSPs). Step 2 selects the top 30 PSPs by ranked median Pearson correlation across proteins. Step 3 implements two aggregation strategies: median-based fusion (Step 3a), yielding QAFIsplit 1.0 and 2.0; and correlation-weighted fusion (Step 3b), yielding QAFIsplit 3.0.

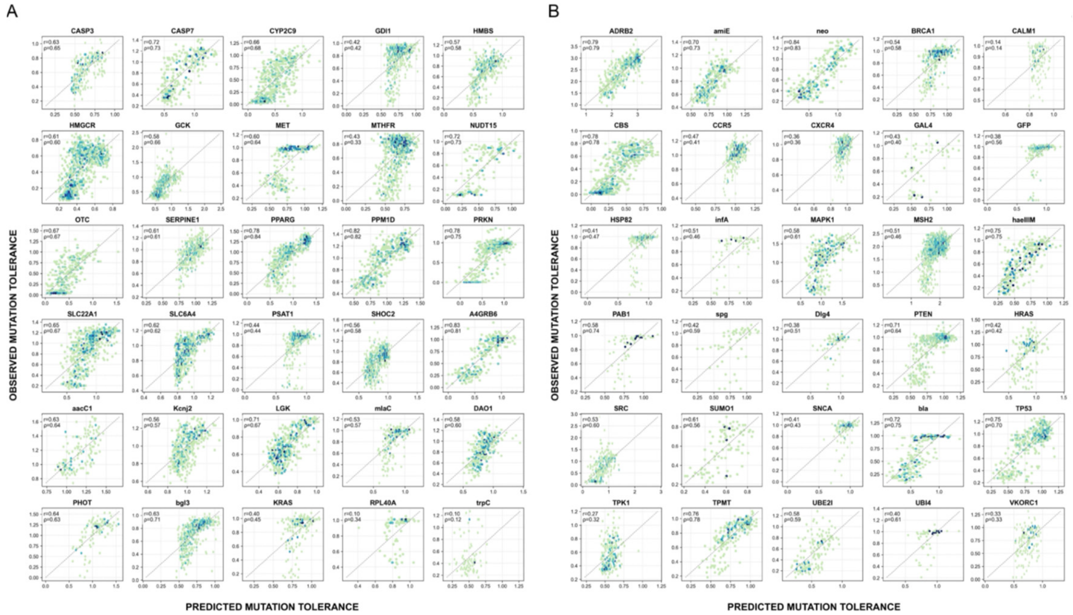

Figure S2. Observed versus predicted mutation tolerance for all 60 proteins in the training set.

As in Figure 2A, for all 60 proteins, shown as density plots. (A) Proteins CASP3 through RPL40A. (B) Proteins ADRB2 through VKORC1. Pearson (r) and Spearman (ρ) correlation coefficients are indicated for each protein; the dashed diagonal indicates perfect agreement. Both observed and predicted mutation tolerance can take slightly negative values at highly intolerant positions, since the zero of the normalized scale is set at the median of the most damaging variants (see Section 4.2).

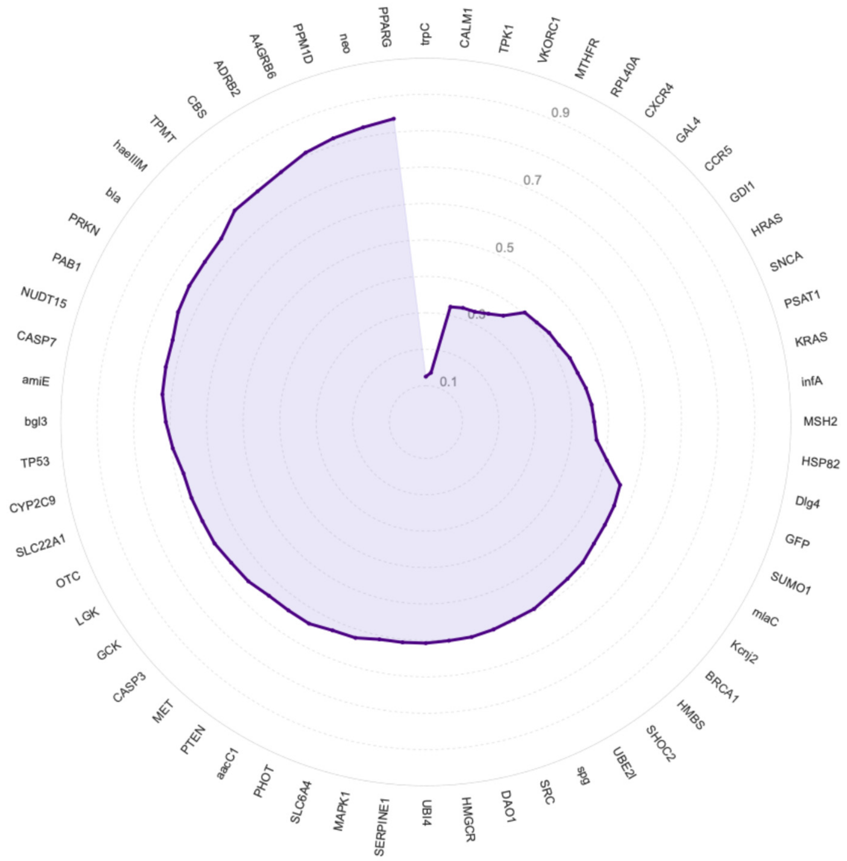

Figure S3. Per-protein Spearman correlations for mutation tolerance prediction across 60 proteins.

Radial plot of per-protein Spearman correlations between predicted and observed mutation tolerance, as described for Figure 2B. Concentric rings indicate correlation values from 0.1 (inner) to 0.9 (outer). Protein names are arranged along the perimeter.

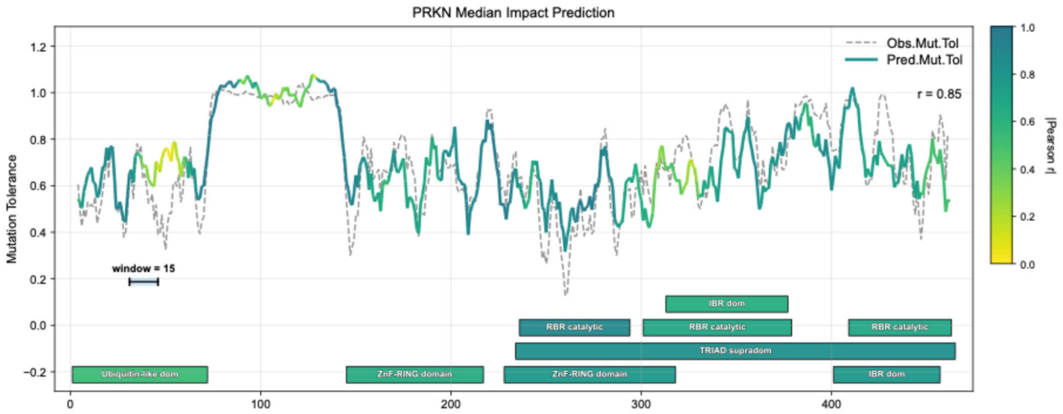

Figure S4. Sequence-level mutation tolerance prediction for PRKN.

Observed (Obs.Mut.Tol; dashed gray line) and predicted (Pred.Mut.Tol; solid line) mutation tolerance plotted along the PRKN protein sequence. The solid line is colored by the local Pearson correlation computed over a sliding window of 15 positions (color scale, right; yellow: low correlation; dark blue: high correlation). Annotated protein domains are shown below the x-axis. Overall Pearson correlation: r = 0.85.

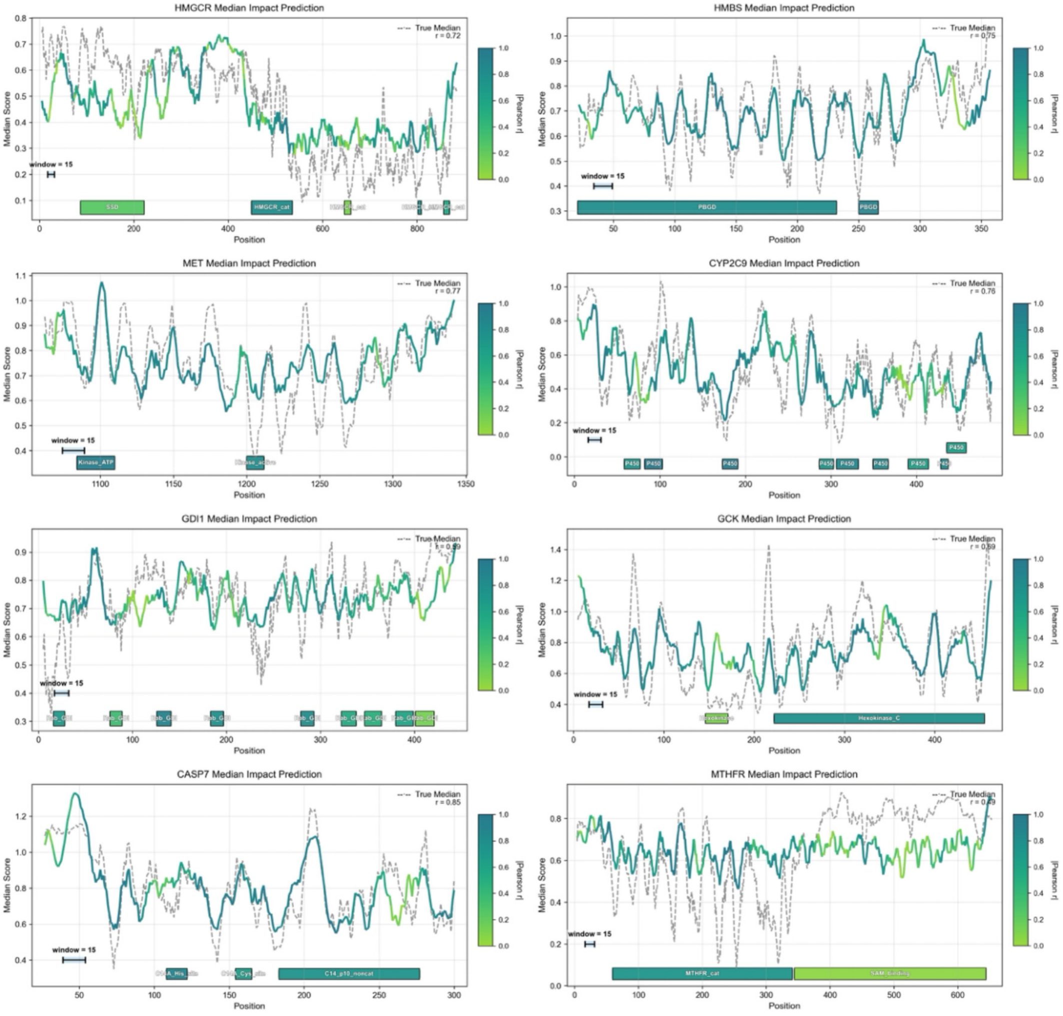

Figure S5. Sequence-level mutation tolerance prediction for eight additional proteins.

As in Figure S4, for HMGCR, HMBS, MET, CYP2C9, GDI1, GCK, CASP7, and MTHFR. Pearson correlation coefficients (r) are shown for each protein.

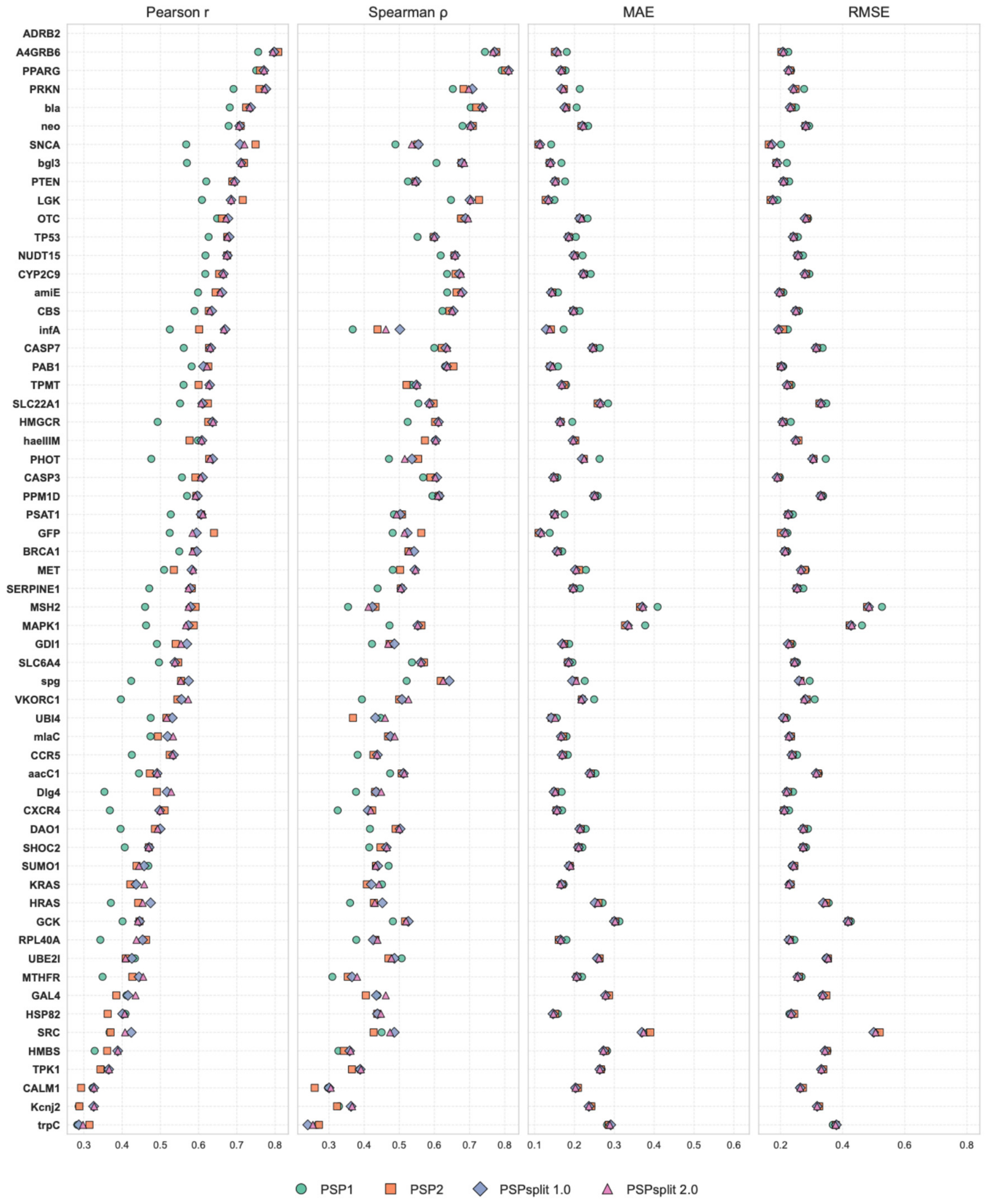

Figure S6. Per-protein performance metrics for all four vePSP models across 60 proteins.

Per-protein Pearson (r), Spearman (ρ), mean absolute error (MAE), and root mean squared error (RMSE) for PSP 1.0 (green circles), PSP 2.0 (orange squares), PSPsplit 1.0 (blue diamonds), and PSPsplit 2.0 (red triangles). Each row represents one protein.

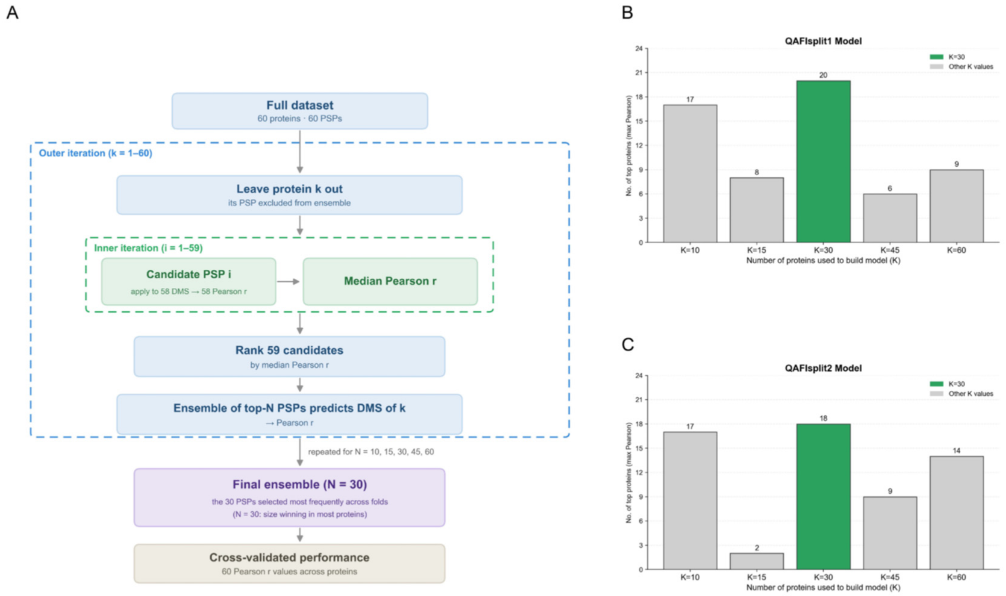

Figure S7. Ensemble size selection procedure and results.

(A) Flowchart of the nested leave-one-protein-out cross-validation used to select ensemble composition and size. For each held-out protein k, the remaining 59 PSPs are ranked by their median Pearson correlation across the other 58 proteins; the top-N PSPs form the ensemble that predicts k. This procedure is repeated for N = 10, 15, 30, 45, and 60. The ensemble size and PSP composition selected most consistently across folds define the final model. (B–C) Number of proteins for which each ensemble size K achieves the highest Pearson correlation, for QAFIsplit 1.0 (B) and QAFIsplit 2.0 (C). The selected size (K = 30, green) outperforms all alternatives in both models.

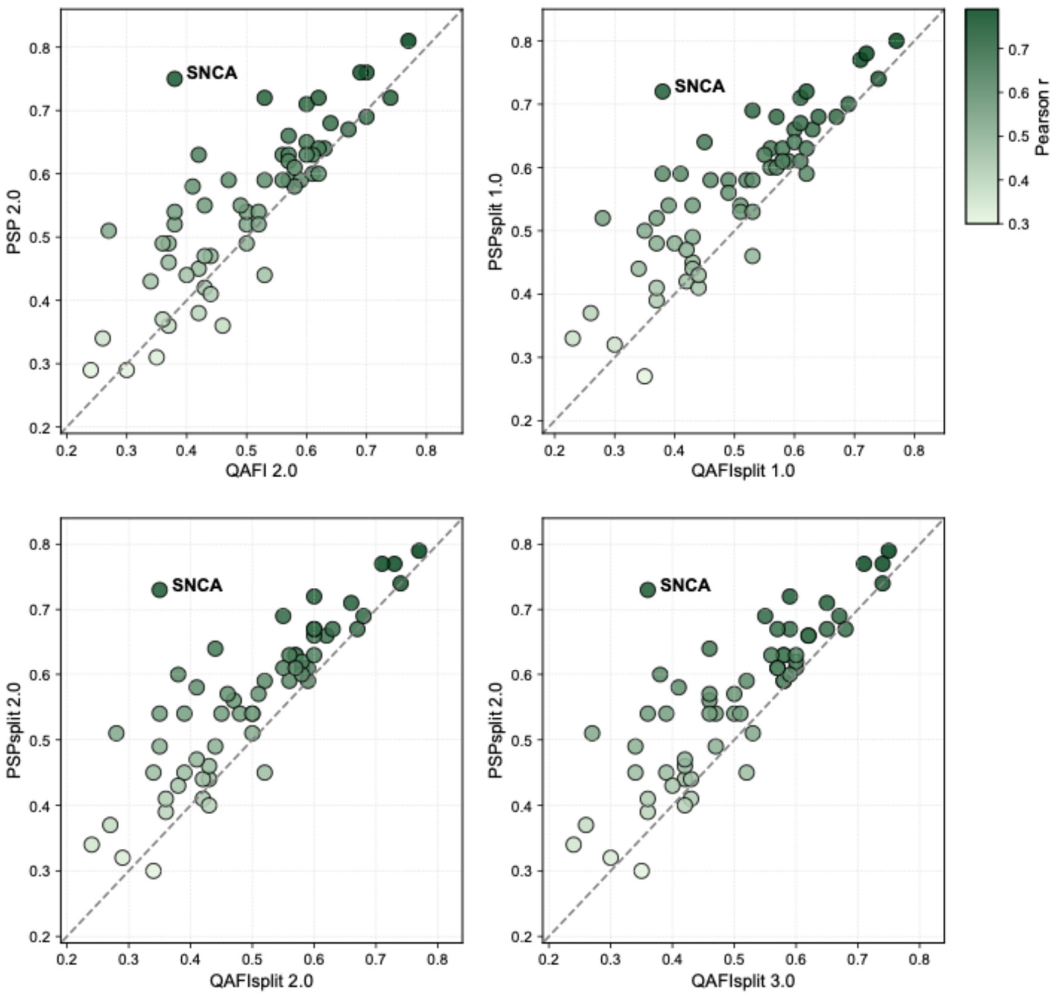

Figure S8. Within-protein (PSP) versus cross-protein (QAFI) Pearson correlations for QAFI models.

As in Figure 4A, for the remaining PSP–QAFI model pairs: PSP 2.0 vs. QAFI 2.0 (top left), PSPsplit 1.0 vs. QAFIsplit 1.0 (top right), PSPsplit 2.0 vs. QAFIsplit 2.0 (bottom left), and PSPsplit 2.0 vs. QAFIsplit 3.0 (bottom right). Each point represents one protein, colored by its PSP Pearson correlation (color scale, top right). The dashed diagonal indicates equal performance; SNCA is labeled in all panels.

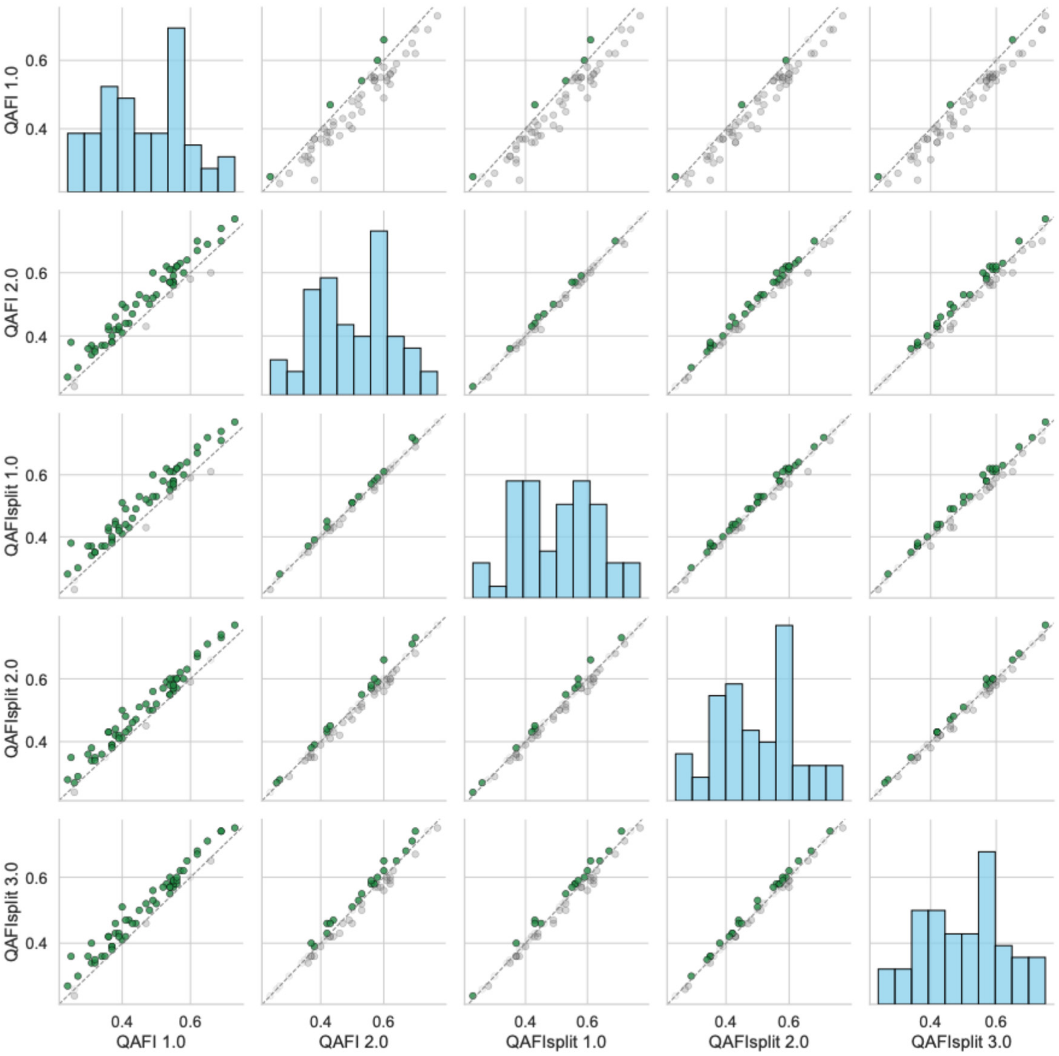

Figure S9. Pairwise comparison of cross-validated Pearson correlations across all five QAFI ensemble models.

As in Figure 3B, for all five QAFI ensemble models: QAFI 1.0, QAFI 2.0, QAFIsplit 1.0, QAFIsplit 2.0, and QAFIsplit 3.0. Diagonal panels show the distribution of per-protein Pearson correlations for each model; off-diagonal panels show pairwise comparisons between models, with the dashed diagonal indicating equal performance.

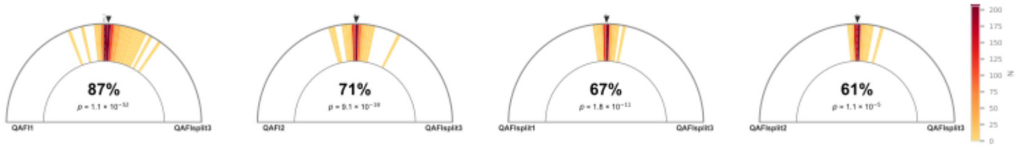

Figure S10. Benchmarking of QAFIsplit 3.0 against other QAFI family members across 402 protein domains.

Circular projection plots comparing QAFIsplit 3.0 against QAFI 1.0, QAFI 2.0, QAFIsplit 1.0, and QAFIsplit 2.0 on the independent Beltrán et al. benchmark (402 protein domains). Plot format and interpretation as described in Figure 5. The color scale (N, right) reflects domain density up to 200 domains per angular bin. See Section 4.19 for details on how these plots are built. P-values are from one-sided exact binomial tests against the null hypothesis that both methods have equal probability of outperforming each other.

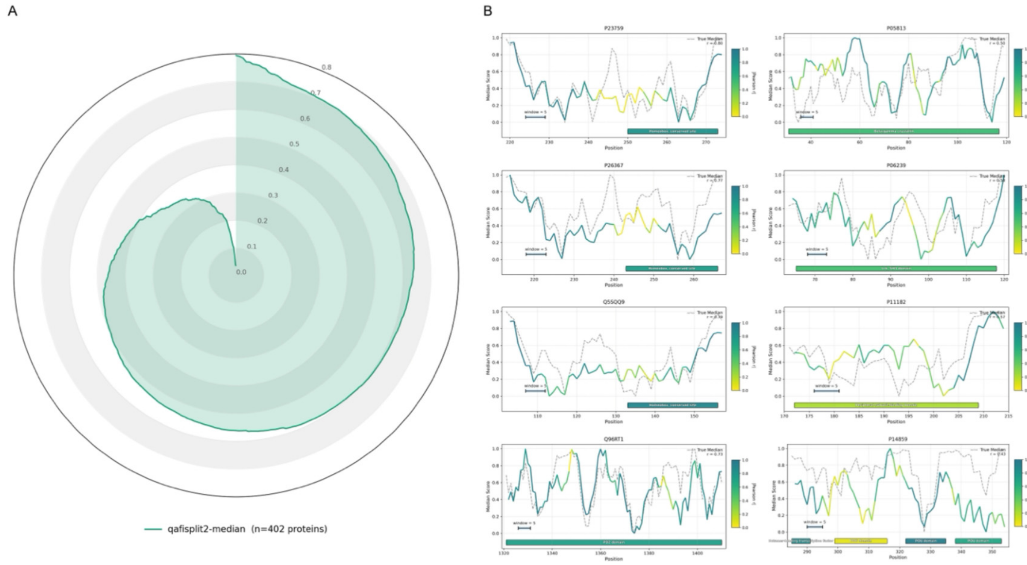

Figure S11. Mutation tolerance prediction performance of QAFImt on the independent Beltrán et al. benchmark.

(A) Radial plot of per-domain Pearson correlations between QAFImt-predicted and experimentally observed positional mutation tolerance across all 402 protein domains. Concentric rings indicate correlation values from 0.0 (inner) to 0.8 (outer). (B) Representative sequence-level profiles for eight protein domains, as described in Figure S4. Observed (True Median; dashed gray) and predicted (solid colored line) mutation tolerance are plotted along the sequence; the solid line is colored by local Pearson correlation over a sliding window of 5 positions. Domain annotations are shown below the x-axis; UniProt accession numbers and Pearson correlation coefficients (r) are indicated for each domain.

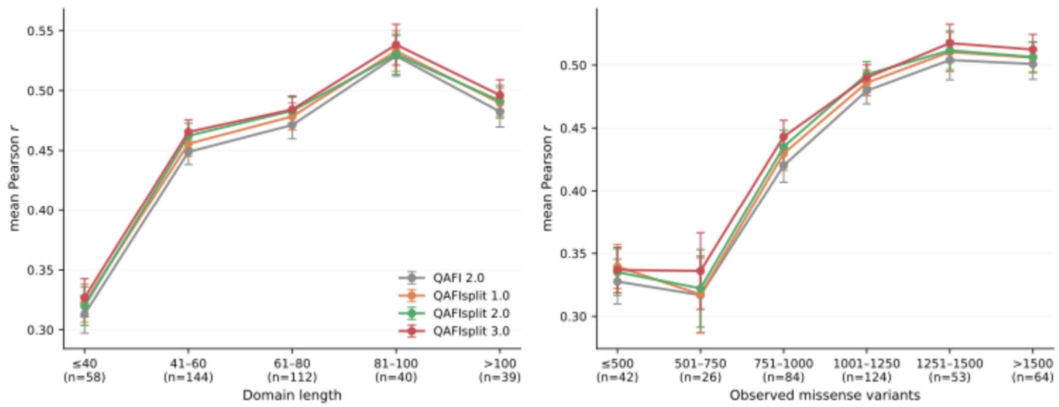

Figure S12. Model performance stratified by domain length and by number of observed missense variants. *Mean per-domain Pearson correlation for QAFI 2.0, QAFIsplit 1.0, QAFIsplit 2.0 and QAFIsplit 3.0 across the 402 domains of the Beltrán et al. benchmark, binned by domain length (left) and by number of measured missense variants (right). Error bars indicate the standard error of the mean; the number of domains per bin is given on the x-axis. QAFIsplit 3.0 performs at or above the other versions in all bins.*

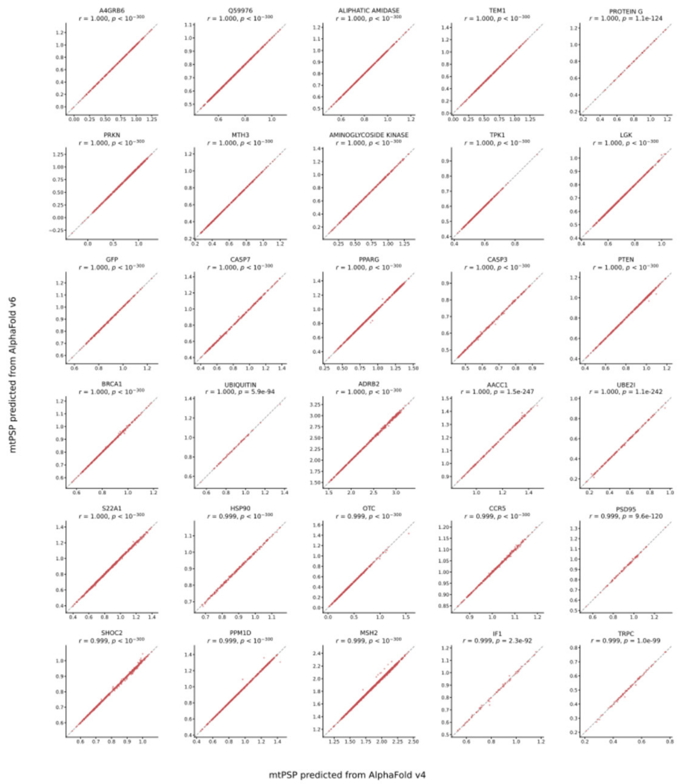
A4GRB6
r = 1.000, $p < 10^{-300}$
Q59976
r = 1.000, $p < 10^{-300}$
ALIPHATIC AMIDASE
r = 1.000, $p < 10^{-300}$
TEM1
r = 1.000, $p < 10^{-300}$
PROTEIN G
r = 1.000, p = 1.1e-124
PRKN
r = 1.000, $p < 10^{-300}$
MTH3
r = 1.000, $p < 10^{-300}$
AMINOGLYCOSIDE KINASE
r = 1.000, $p < 10^{-300}$
TPK1
r = 1.000, $p < 10^{-300}$
LGK
r = 1.000, $p < 10^{-300}$
GFP
r = 1.000, $p < 10^{-300}$
CASP7
r = 1.000, $p < 10^{-300}$
PPARG
r = 1.000, $p < 10^{-300}$
CASP3
r = 1.000, $p < 10^{-300}$
PTEN
r = 1.000, $p < 10^{-300}$
BRCA1
r = 1.000, $p < 10^{-300}$
UBIQUITIN
r = 1.000, p = 5.9e-94
ADRB2
r = 1.000, $p < 10^{-300}$
AACC1
r = 1.000, p = 1.5e-247
UBE2I
r = 1.000, p = 1.1e-242
S22A1
r = 1.000, $p < 10^{-300}$
HSP90
r = 0.999, $p < 10^{-300}$
OTC
r = 0.999, $p < 10^{-300}$
CCR5
r = 0.999, $p < 10^{-300}$
PSD95
r = 0.999, p = 9.6e-120
SHOC2
r = 0.999, $p < 10^{-300}$
PPM1D
r = 0.999, $p < 10^{-300}$
MSH2
r = 0.999, $p < 10^{-300}$
IF1
r = 0.999, p = 2.3e-92
TRPC
r = 0.999, p = 1.0e-99
mtPSP predicted from AlphaFold v6
mtPSP predicted from AlphaFold v4

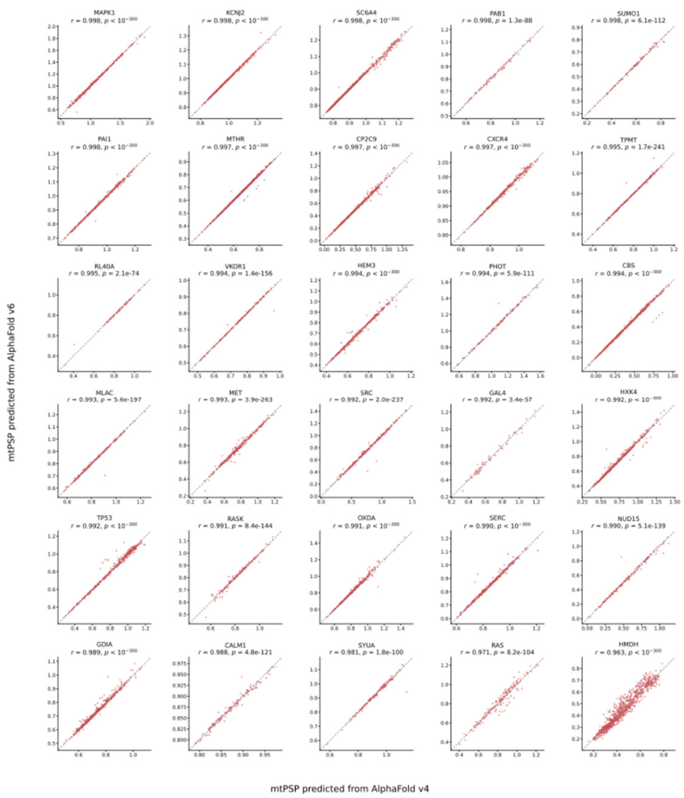
MAPK1
r = 0.998, $p < 10^{-300}$
KCNJ2
r = 0.998, $p < 10^{-300}$
SC6A4
r = 0.998, $p < 10^{-300}$
PAB1
r = 0.998, p = 1.3e-88
SUMO1
r = 0.998, p = 6.1e-112
PAI1
r = 0.998, $p < 10^{-300}$
MTHR
r = 0.997, $p < 10^{-300}$
CP2C9
r = 0.997, $p < 10^{-300}$
CXCR4
r = 0.997, $p < 10^{-300}$
TPMT
r = 0.995, p = 1.7e-241
RL40A
r = 0.995, p = 2.1e-74
VKOR1
r = 0.994, p = 1.4e-156
HEM3
r = 0.994, $p < 10^{-300}$
PHOT
r = 0.994, p = 5.9e-111
CBS
r = 0.994, $p < 10^{-300}$
MLAC
r = 0.993, p = 5.6e-197
MET
r = 0.993, p = 3.9e-263
SRC
r = 0.992, p = 2.0e-237
GAL4
r = 0.992, p = 3.4e-57
HXK4
r = 0.992, $p < 10^{-300}$
TP53
r = 0.992, $p < 10^{-300}$
RASK
r = 0.991, p = 8.4e-144
OXDA
r = 0.991, $p < 10^{-300}$
SERC
r = 0.990, $p < 10^{-300}$
NUD15
r = 0.990, p = 5.1e-139
GDIA
r = 0.989, $p < 10^{-300}$
CALM1
r = 0.988, p = 4.8e-121
SYUA
r = 0.981, p = 1.8e-100
RAS
r = 0.971, p = 8.2e-104
HMDH
r = 0.963, $p < 10^{-300}$
mtPSP predicted from AlphaFold v6
mtPSP predicted from AlphaFold v4

Figure S13. Effect of the AlphaFold database release on predicted mutation tolerance. *mtPSP predictions computed from AlphaFold v6 structures (y-axis) versus the v4 structures used throughout this study (x-axis), for all 60 training proteins. Each point is one position; the dashed diagonal indicates perfect agreement. Proteins are sorted by Pearson correlation, shown with its p-value for each protein. Agreement is essentially complete throughout (r from 0.963 to 1.000, median 0.999), indicating that predicted mutation tolerance is essentially insensitive to the database update.*

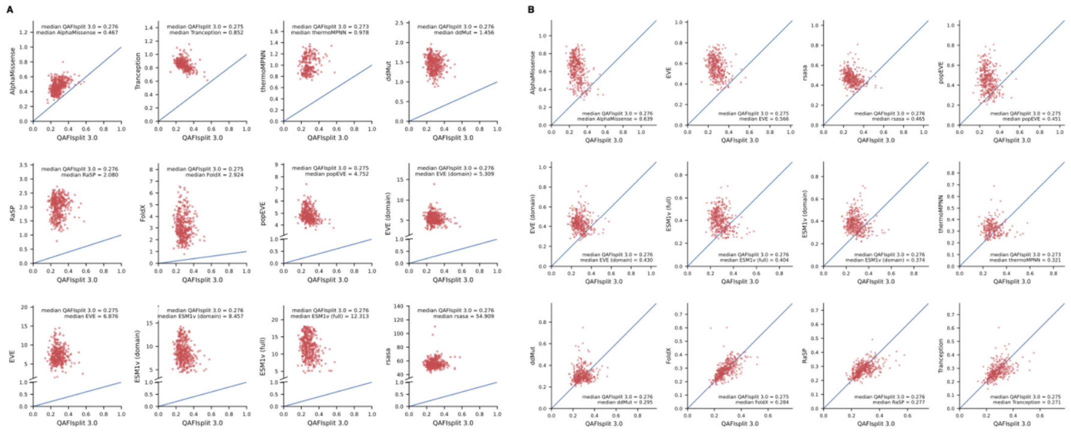

Figure S14. Deviation from experimental values, before and after rescaling the output of each method. *Root mean squared error per domain between predicted and observed normalized DMS scores, for QAFIsplit 3.0 (x-axis) and each of the methods compared (y-axis), across the 402 domains of the Beltrán et al. benchmark. Methods whose output increases with loss of function were inverted beforehand, and the experimental scores were left unmodified throughout.* ***(A)*** *Raw output of each method, with no further transformation. Note the differing y-axis scales: most methods return quantities on scales unrelated to the normalized functional scale, so their deviation reflects this difference in units rather than predictive error.* ***(B)*** *After rescaling the output of each method to [0, 1] within each domain, a transformation not part of the original methods. Median deviations for both methods are shown in each panel. The diagonal indicates equal deviation in both panels.*

SUPPLEMENTARY TABLES

Table S1. Training details and model specifications of the PSP models.

Table S2. Proteins in the QAFI ensembles (N = 30). QAFIsplit 3.0 combines the same 30 proteins by correlation-weighted fusion.

Table S3. Model specifications of the QAFI models and the mapping between QAFI and PSP models.

Table S4. Full protein list and metadata for the 60 DMS datasets. Coverage of the full protein = variants / (length × 19); coverage of the scanned region = variants / (positions covered × 19).

Table S5. Normalized variant-level scores for all 60 datasets. A normalized score of 1 corresponds to no detectable impact on protein function.

Table S6. Full definitions of the features used by the QAFI models.

Table S7. The neco matrices. Rows are the native residue, columns the mutant residue; higher values indicate substitutions more compatible with the local evolutionary context.
